# Supplementary material for: Targeting Endogenous K-RAS for Degradation through the Affinity-Directed Protein Missile System
Source: Cell Chem Biol. 2020 Sep 17;27(9):1151–1163.e6. doi: 10.1016/j.chembiol.2020.06.012 (PMC7505679; doi:10.1016/j.chembiol.2020.06.012)
Supplement: Document S1. Figures S1–S6 [file mmc1.pdf]

Cell Chemical Biology, Volume *27*

Supplemental Information

Targeting Endogenous K-RAS for Degradation through the Affinity-Directed Protein Missile System

Sascha Röth, Thomas J. Macartney, Agnieszka Konopacka, Kwok-Ho Chan, Houjiang Zhou, Markus A. Queisser, and Gopal P. Sapkota

A

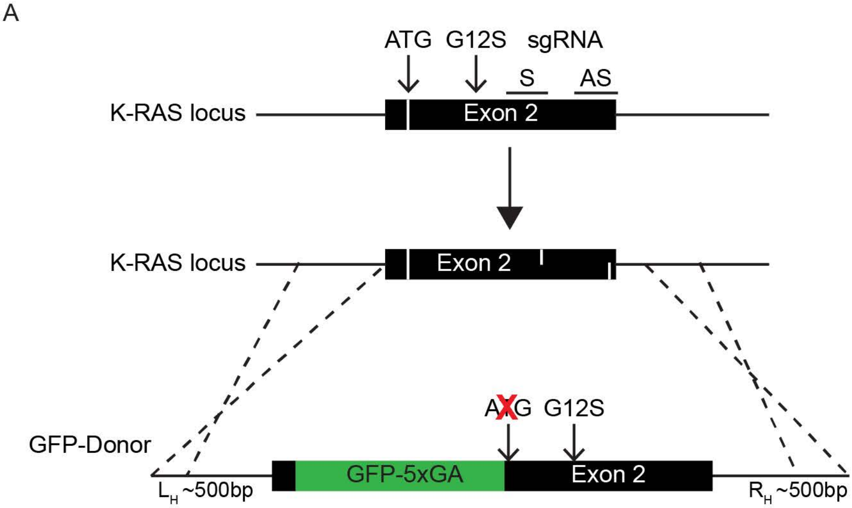

B

Codon	10	11	12	13
AA	Gly	Ala	Gly	Gly
WT	GGT	GCA	GGA	GGT
Donor	GGA	GCT	TCT	GGC
AA	Gly	Ala	Ser	Gly

C

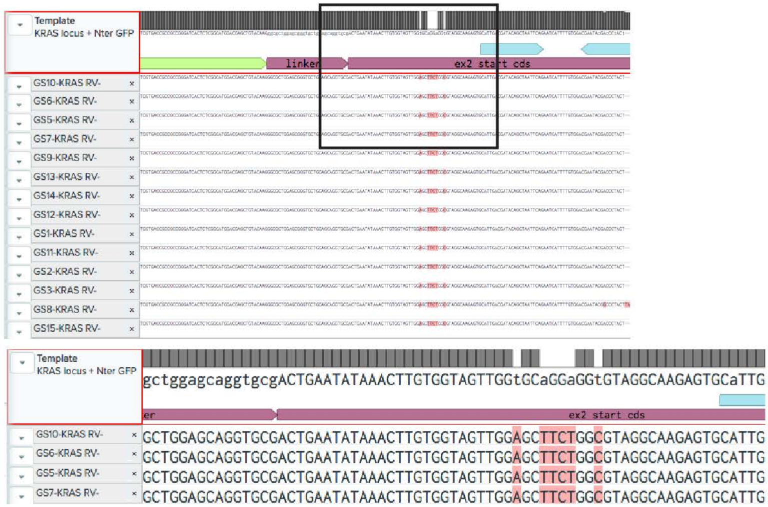

Figure S1 - Characterization of A549^{GFPKRAS} - Related to Figure 1

(A) Schematic representation of the CRISPR/Cas9 strategy used for A549 cells. Two plasmids encoding sgRNA sequences targeting the K-RAS locus on exon 2 were co-expressed with Cas9-D10A, to create two nicks in K-RAS complementary strands for a double stranded break. A donor plasmid consisting of GFP cDNA sequence without the stop codon followed by a GAGAGAGAGA linker flanked by Left and Right homology arms (L_H and R_H, respectively) was designed and co-transfected to allow homologous recombination for insertion of the GFP-5xGA tag onto the native K-RAS locus at the start codon. Consequently, the start codon of K-RAS was eliminated. (B) The indicated silent mutations on sgRNA target codons (10-13) were introduced in the donor sequence to block subsequent dsDNA breaks following integration of the donor on K-RAS locus. (C) A screenshot of DNA sequence analysis from Benchling of the resulting GFP-positive clone. Top: 14 DNA sequence files were aligned against the predicted WT RAS gene locus sequence with the GFP-fusion (indicated in light green) and the 5xGA linker. The box indicates area of the image that is magnified below. The magnified area shows DNA sequence alignment of the A549^{GFPKRAS} cell line at the site of the G12S mutation, which also shows silent mutations. The Ab1 files containing the DNA sequence chromatograms are deposited online.

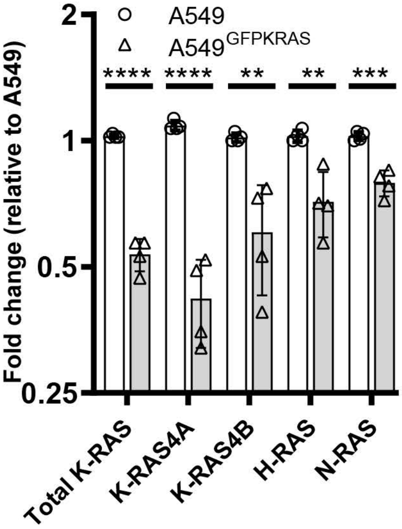

Figure S2 - Analysis of RAS transcript levels in A549 WT and A549[GFPKRAS] - Related to Figure 1

mRNA was extracted from indicated cell lines and cDNA was synthesised. Fold changes in RAS expression were calculated after qRT-PCR with specific primers between A549[GFPKRAS] (grey bars) and A549 WT (white bars). Error bars (SD) are shown for n=4. Statistical significance was calculated with an unpaired, parametric, two-tailed t-test. $p<0.01$ = **; $p<0.001$ = ***; $p<0.0001$ = ****.

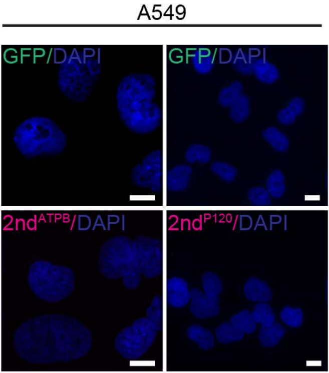

Figure S3 - Negative controls for Figure 1D

Experimental procedure is described in Figure 1D. An additional slide of A549 WT cells (GFP negative) was treated with the secondary antibody used for ATPB or P120. Exposure times are set to be the same as for the positive sample slides in Figure 1D. Scalebar = 10μm

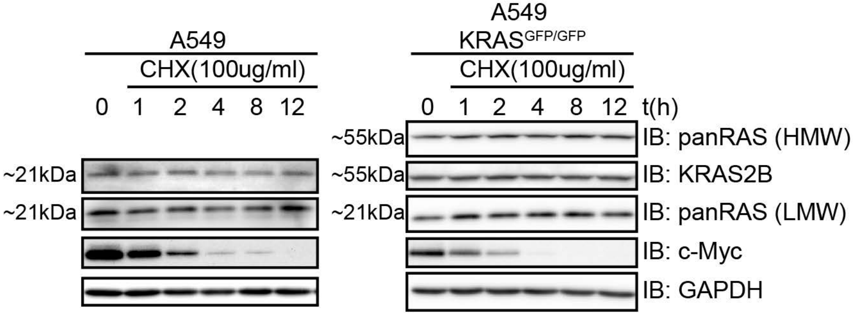

Figure S4 - Representative blots of quantification in Fig. 1E.

Samples were treated as described in Fig. 1E, run on an SDS-PAGE and blotted onto PVDF membrane. The indicated antibodies were used for detection of protein levels. Shown are representative blots for the quantification in Fig. 1E.

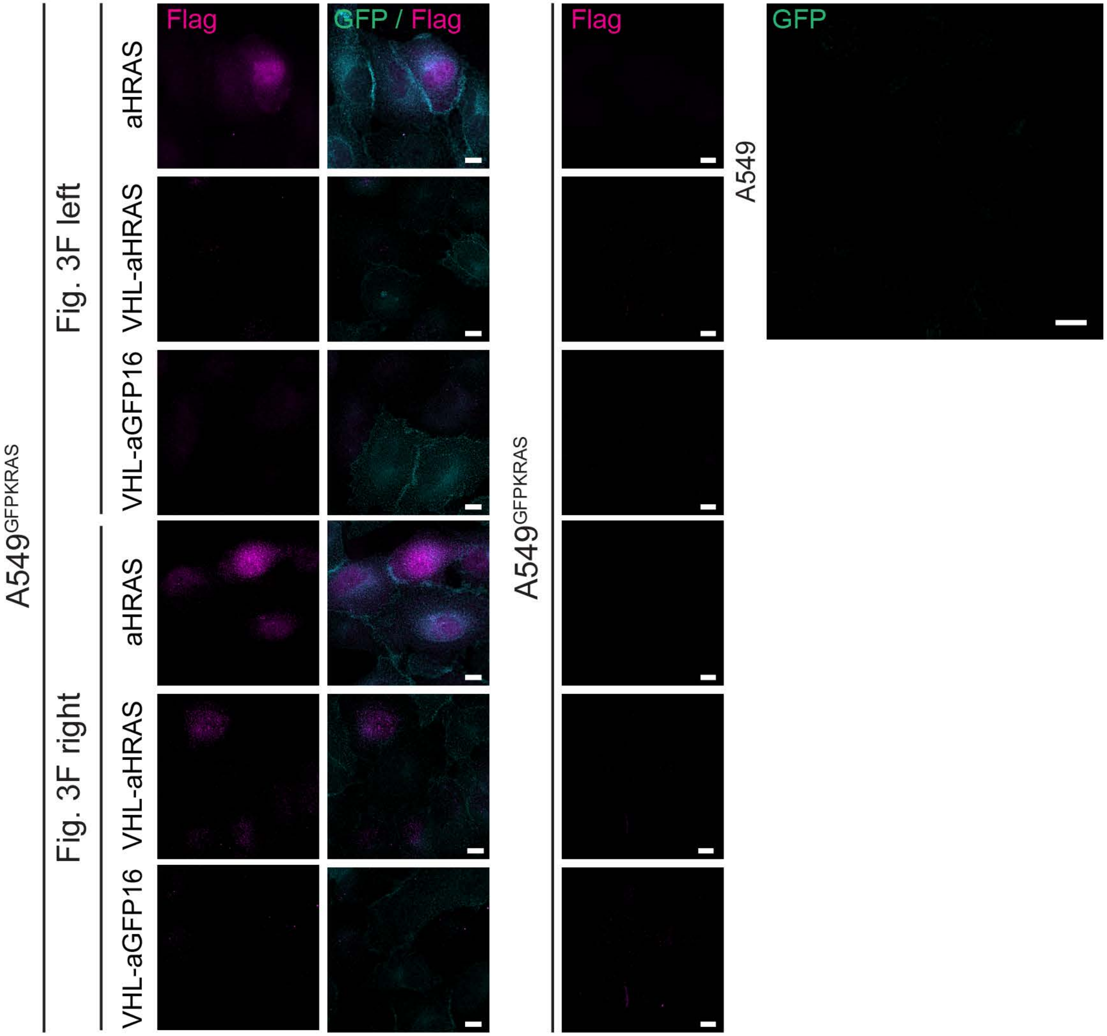

Figure S5 - FLAG signal and controls for Figure 3F.

Slides of Fig. 3F (A549^{GFPKRAS} with or without indicated transductions) were additionally stained with anti-FLAG antibody and secondary stain for detection in the 594 channel (Flag) (2 leftmost columns). The top 3 rows represent the left column of Fig. 3F, the bottom 3 rows the right column of Fig. 3F. Appropriate negative control (A549^{GFPKRAS} cells without a FLAG construct) were stained with the same antibody combination and exposure time (Column 3). Brightness and contrast for individual positive stains were background adjusted for these samples (note that the same negative sample is used for more than one picture). Additionally A549 WT cells were stained with the GFP antibody to act as negative control for GFP signal in main Figure 3F (panel on the right). Scalebar = 10μm.

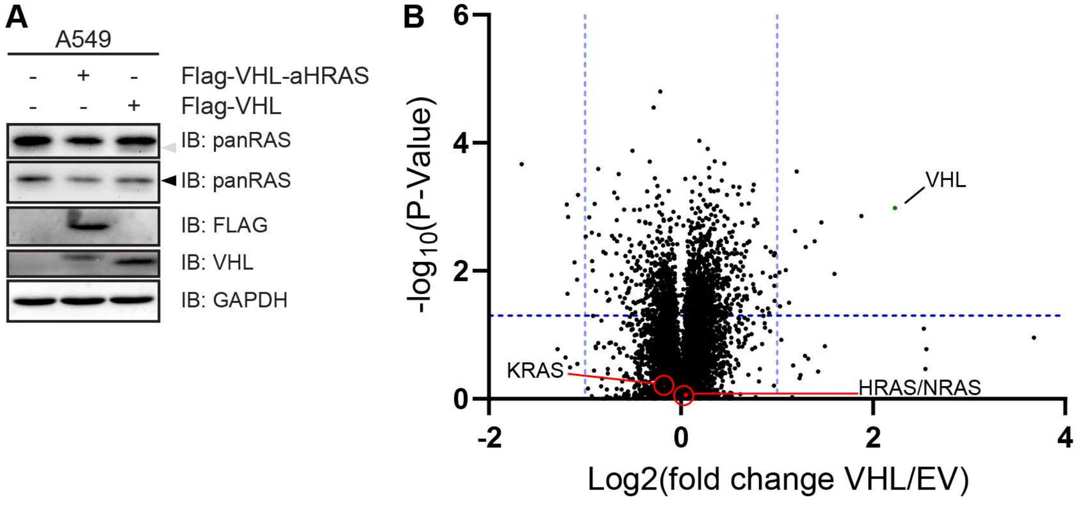

Figure S6 - Proteomic analysis of AdPROM - Related to Figure 4D

(A) Pooled lysates of triplicate A549 cells transduced with and selected for indicated plasmids were subjected to SDS-PAGE and Western blot analysis with indicated antibodies, used for TMT labelling and total proteome analysis. Two images are shown for panRAS detection to highlight K-RAS (black arrowhead) or H-/N/RAS (grey arrowhead) signals. (B) Volcano plot on proteins significantly down- or up-regulated by transduction of A549 cells with Flag-VHL compared to pBabeD empty vector. Horizontal line shows significance level of p=0.05. Vertical lines show 2 fold change. Positions of KRAS, HRAS, NRAS and VHL are indicated.
