# Supplementary material for: Targeting Endogenous K-RAS for Degradation through the Affinity-Directed Protein Missile System
Source: Cell Chem Biol. 2020 Sep 17;27(9):1151–1163.e6. doi: 10.1016/j.chembiol.2020.06.012 (PMC7505679; doi:10.1016/j.chembiol.2020.06.012)
Supplement: Document S2. Article plus Supplemental Information [file mmc4.pdf]

Article

Cell Chemical Biology

Targeting Endogenous K-RAS for Degradation through the Affinity-Directed Protein Missile System

Highlights

- Generation of A549 cells with a homozygous knockin of GFP tag on the *KRAS* gene
- Proteasomal degradation of endogenous GFP-K-RAS using a VHL-GFP-nanobody fusion
- Proteasomal degradation of endogenous H/K-RAS using VHL-H/K-RAS-monobody fusion

Authors

Sascha Röth, Thomas J. Macartney, Agnieszka Konopacka, Kwok-Ho Chan, Houjiang Zhou, Markus A. Queisser, Gopal P. Sapkota

Correspondence

g.sapkota@dundee.ac.uk

In Brief

By using the affinity-directed protein missile (AdPROM) system comprised of VHL and high-affinity binders of GFP and H/K-RAS, Röth et al. demonstrate the targeted proteasomal degradation of GFP-K-RAS, which was knocked in using CRISPR/Cas9, and endogenous H/K-RAS.

Röth et al., 2020, Cell Chemical Biology *27*, 1151–1163
September 17, 2020 © 2020 The Authors. Published by Elsevier Ltd.
https://doi.org/10.1016/j.chembiol.2020.06.012

Cell Chemical Biology

CellPress
OPEN ACCESS

Article

Targeting Endogenous K-RAS for Degradation through the Affinity-Directed Protein Missile System

Sascha Röth,[1] Thomas J. Macartney,[1] Agnieszka Konopacka,[2] Kwok-Ho Chan,[2] Houjiang Zhou,[1] Markus A. Queisser,[2] and Gopal P. Sapkota[1,3,*]
[1]Medical Research Council Protein Phosphorylation and Ubiquitylation Unit, University of Dundee, Dundee, UK
[2]GlaxoSmithKline, Protein Degradation Group, Medicines Research Centre, Gunnels Wood Road, Stevenage, UK
[3]Lead Contact
*Correspondence: g.sapkota@dundee.ac.uk
https://doi.org/10.1016/j.chembiol.2020.06.012

SUMMARY

K-RAS is known as the most frequently mutated oncogene. However, the development of conventional K-RAS inhibitors has been extremely challenging, with a mutation-specific inhibitor reaching clinical trials only recently. Targeted proteolysis has emerged as a new modality in drug discovery to tackle undruggable targets. Our laboratory has developed a system for targeted proteolysis using peptidic high-affinity binders, called "AdPROM." Here, we used CRISPR/Cas9 technology to knock in a GFP tag on the native *K-RAS* gene in A549 adenocarcinoma (A549^{GFPKRAS}) cells and constructed AdPROMs containing high-affinity GFP or H/K-RAS binders. Expression of GFP-targeting AdPROM in A549^{GFPKRAS} led to robust proteasomal degradation of endogenous GFP-K-RAS, while expression of anti-HRAS-targeting AdPROM in different cell lines resulted in the degradation of both GFP-tagged and untagged K-RAS, and untagged H-RAS. Our findings imply that endogenous RAS proteins can be targeted for proteolysis, supporting the idea of an alternative therapeutic approach to these undruggable targets.

INTRODUCTION

The three RAS oncogenes, *H-RAS*, *K-RAS*, and *N-RAS*, represent the most frequently mutated genes in cancer (Cox et al., 2014; Hobbs et al., 2016). They encode four highly similar proteins, namely H-RAS, N-RAS, K-RAS4A, and K-RAS4B, which undergo C-terminal farnesylation (Reiss et al., 1990; Schaber et al., 1990). Farnesylation, in combination with palmitoylation in the hypervariable region (HVR) (N-RAS, H-RAS, and K-RAS4A) or with a polybasic signal in the HVR (K-RAS4B), mediates the plasma membrane interaction (Ahearn et al., 2012). RAS proteins are small GTPases, which cycle between the GTP-bound (active) and GDP-bound (inactive) states, controlled by guanosine nucleotide exchange factors and GTPase activating proteins (GAPs) (Vigil et al., 2010). Activation of RAS proteins by various extracellular growth factors initiates activation of numerous downstream signaling networks, including BRAF/mitogen-activated protein kinase (MAPK) and phosphatidylinositol 3-kinase pathways (Khan et al., 2019a), which are critical for cell proliferation and viability. Many pathogenic mutations in *RAS* genes impair GAP-mediated GTP hydrolysis, thereby favoring the persistence of the active RAS-GTP state, which triggers constitutive activation of downstream signaling resulting in unchecked proliferation of cancer cells (Hobbs et al., 2016; Marcus and Mattos, 2015).

As the oncogenicity of RAS mutations has been known for over three decades, intensive efforts have been made toward drugging them. These efforts are yet to result in effective RAS-inhibitor therapies (Cox et al., 2014; Papke and Der, 2017). This has promoted the perception that RAS proteins are undruggable. Several factors make RAS proteins difficult targets to engineer selective small-molecule inhibitors. First, the relatively high concentrations of GTP and GDP in cells and picomolar affinity to binding RAS proteins makes it almost impossible to develop GTP/GDP analogs as inhibitors (Cox et al., 2014; John et al., 1990). Second, structural analysis of RAS proteins revealed few sufficiently large and deep hydrophobic pockets on the surface for small-molecule binding (O'Bryan, 2019; Pai et al., 1989). Recently, a covalent inhibitor targeting a cysteine in K-RAS G12C was developed to target this specific mutation (Ostrem et al., 2013). However, these barriers and failure to directly target RAS have prompted researchers to explore targeting upstream regulators, or downstream effectors of RAS proteins (Cox et al., 2014; Kang et al., 2009; Leung et al., 2018; Papke and Der, 2017; Waldmann et al., 2004), as well as altering levels of RAS protein, for example, by inducing targeted degradation of RAS (Nabet et al., 2018).

Most targeted protein degradation approaches harness the cellular proteolytic pathways that naturally maintain proteostasis, with the ubiquitin proteasome system (UPS) being frequently exploited (Röth et al., 2019). Protein degradation by the UPS is triggered by conjugation of ubiquitin chains onto the target protein, which is achieved through a sequential action of three enzymes: the ubiquitin-activating enzyme (E1), which activates

This is an open access article under the CC BY license (http://creativecommons.org/licenses/by/4.0/).

the C-terminal glycine residue of ubiquitin in an ATP-dependent manner; a ubiquitin-conjugating enzyme (E2), which conjugates the activated ubiquitin to its active site cysteine; and a ubiquitin ligase (E3), which facilitates the transfer of ubiquitin from E2 to primarily lysine residues on substrate proteins (Pickart and Eddins, 2004; Roos-Mattjus and Sistonen, 2004). Further ubiquitylation on one or more lysine residues within ubiquitin then triggers polyubiquitylation, followed by degradation by the proteasome (Akutsu et al., 2016; Komander and Rape, 2012; Yau and Rape, 2016). Targeting RAS for proteolysis relies on the engagement of the cellular proteolytic systems for its ubiquitylation and degradation. In this context, it has been shown that the heterobifunctional molecule dTAG-13, which recruits FKBP12^{F36V}-tagged proteins of interest (POIs) to the CRBN/CUL4A E3 ubiquitin ligase for their degradation, can degrade FKBP12^{F36V}-KRAS^{G12V} overexpressed in cell lines (Nabet et al., 2018). However, FKBP12^{F36V} itself can be targeted for ubiquitylation when using heterobifunctional small-molecule binders (Winter et al., 2015). Therefore, it remains unclear, whether using dTAG13 on FKBP12^{F36V}-K-RAS results in the ubiquitination of K-RAS or FKBP12^{F36V}. Such information is not only key to evaluate proteolysis as a druggable approach for targeting RAS proteins but also to inform on the development of effective heterobifunctional RAS degraders.

We have previously developed an effective proteolytic affinity-directed protein missile (AdPROM) system for UPS-mediated POI degradation (Fulcher et al., 2016, 2017). AdPROM consists of a fusion of von Hippel-Lindau (VHL) protein, a substrate recruiter of the CUL2-RING E3 ligase complex, and high-affinity binders, such as nanobodies and monobodies, of POIs. Delivering AdPROM into multiple cell lines through retroviral transductions led to efficient degradation of endogenous target proteins, including SHP2 and ASC (Fulcher et al., 2017). Furthermore, to target POIs for which no high-affinity polypeptide binders exist, we utilized CRISPR/Cas9 genome editing to rapidly introduce GFP tags on endogenous VPS34 and PAWS1 genes, and used the AdPROM system consisting of anti-GFP nanobody fused to VHL to achieve near complete degradation of the endogenous GFP-VPS34 and PAWS1-GFP proteins (Fulcher et al., 2016). In this study, we explore the use of the AdPROM system, and demonstrate its efficacy, for targeted degradation of endogenously GFP-tagged K-RAS and untagged, endogenous K-RAS from cells.

RESULTS

Generation of a GFP-KRAS Knockin Non-small Cell Lung Cancer A549 Cell Line

The high degree of amino acid sequence similarity between the four RAS proteins, i.e., K-RAS4A, K-RAS4B, H-RAS, and N-RAS (Figure 1A), and the subsequent difficulty in generating selective antibodies against individual isoforms pose substantial challenges in studying specific RAS proteins (Waters et al., 2017). To explore targeted proteolysis of K-RAS using the AdPROM system, we used CRISPR/Cas9 technology to generate an A549 non-small cell lung carcinoma (NSCLC) cell line harboring a homozygous knockin of green fluorescent protein (GFP) cDNA at the N terminus of the native *K-RAS* gene (Figure S1). As K-RAS4A and K-RAS4B are splice variants differing only in their extreme C terminus (Figure 1A), this approach allowed us to simultaneously tag both isoforms with GFP. The homozygous GFP knockins on the native KRAS locus (A549^{GFPKRAS}) were verified by genomic sequencing (Figure S1). Moreover, by western blot analysis using both panRAS and K-RAS4B antibodies, the appearance of higher-molecular-weight GFP-K-RAS species with a concurrent disappearance of the native-molecular-weight K-RAS species was evident in the A549^{GFPKRAS} cell line compared with wild-type (WT) A549 control cells (Figure 1B). The use of a panRAS antibody resulted in the detection of two distinct bands in A549 WT cells (Figure 1B). As the lower band remained intact in A549^{GFPKRAS} cells, it most likely corresponds to H- and/or N-RAS (Figure 1B). However, in A549 cells we were unable to detect any endogenous signals with most commercially available H-RAS-, N-RAS-, or K-RAS4A-specific antibodies (listed in the STAR Methods). As K-RAS is an integral part of the MAP kinase signaling pathway, we wanted to analyze the effect GFP fusion would have on the MAPK signaling pathways. Under cell culture conditions, both MEK1/2 and ERK phosphorylations were strongly decreased in A549^{GFPKRAS} cells compared with A549 WT cells, while levels of BRAF were slightly decreased (Figure 1B). Interestingly, phosphorylation of EGF receptor at Tyr1068 and AKT phosphorylation at Ser473 was higher in A549^{GFPKRAS} cells than in WT cells (Figure 1B). By qRT-PCR, we showed that levels of H- and N-RAS transcripts were slightly reduced in A549^{GFPKRAS} cells compared with WT A549 cells, while transcript levels of K-RAS were reduced by roughly 50% (Figure S2). We were able to efficiently immunoprecipitate GFP-K-RAS from A549^{GFPKRAS} but not WT A549 cell extracts (Figure 1C).

A number of RAS antibodies have been evaluated for selective recognition of the different RAS proteins by western blotting (Waters et al., 2017), but none of these have been selective for use in immunofluorescence studies. Consequently, studies evaluating subcellular distribution of RAS proteins have been restricted to overexpression systems. Validation of A549^{GFPKRAS} cells allowed us to investigate the subcellular distribution of endogenous GFP-K-RAS driven by the native promoter. Endogenous GFP-K-RAS displayed predominantly plasma membrane distribution, which was confirmed by co-staining with P120 catenin, which is known to localize to the plasma membrane (Reynolds et al., 1994) (Figures 1D and S3). In addition, we also observed some weak cytoplasmic localization of GFP-K-RAS. However, no co-localization of GFP-K-RAS was observed with mitochondrial marker ATPB (Schatz and Butow, 1983) (Figures 1D and S3).

Finally, we compared turnover of WT K-RAS and GFP-K-RAS proteins by adding cycloheximide to the respective cell line and analyzing protein levels over the course of 12 h. We could not find any remarkable differences in protein stability between WT and GFP-K-RAS (Figures 1E and S4). However, as expected, a robust degradation of c-myc was observed within 2–4 h (Figure S4).

Targeted Degradation of GFP-K-RAS by the Proteolytic AdPROM System

We sought to test whether endogenously expressed GFP-K-RAS protein in A549^{GFPKRAS} cells could be targeted for degradation by AdPROM (Fulcher et al., 2016, 2017). We have previously shown

CellPress
OPEN ACCESS

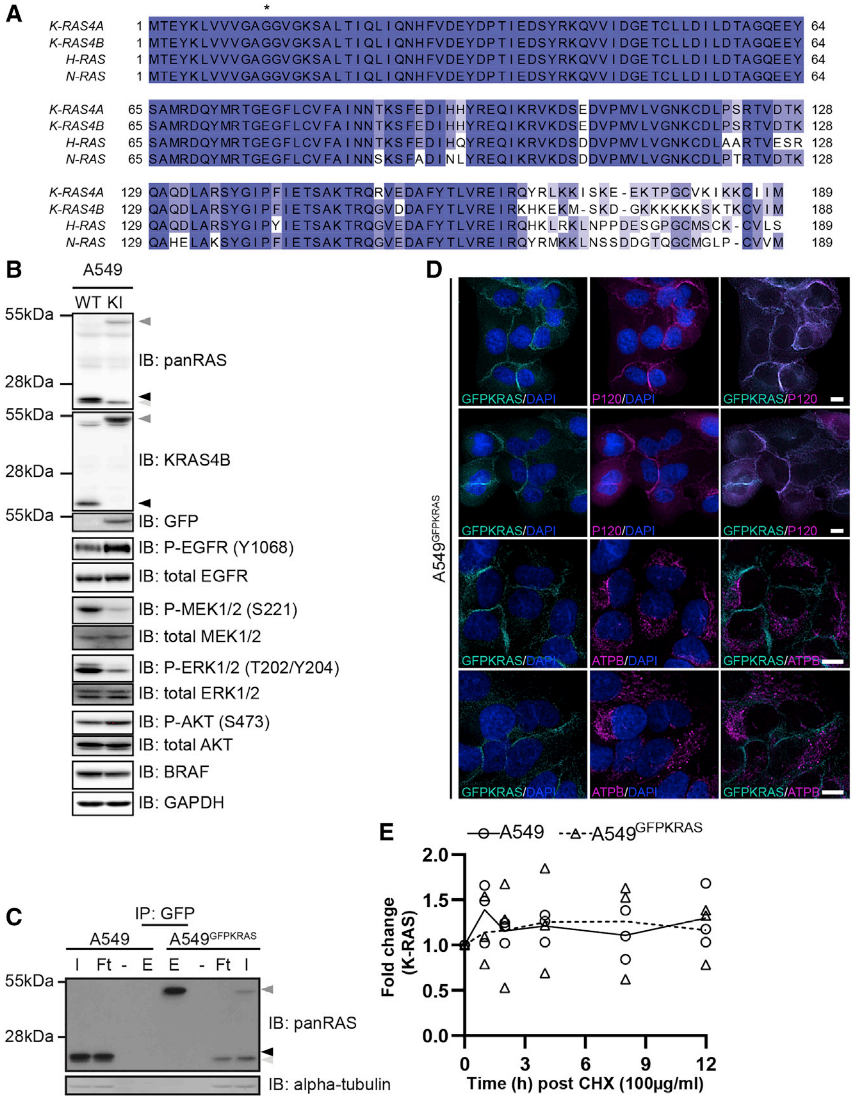

Figure 1. Generation of GFP-K-RAS Knockin in A549 NSCLC Cells by CRISPR/Cas9

(A) Sequence Alignment of RAS protein isoforms K-RAS4A (UniProt: P01116-1), K-RAS4B (Uniprot: P01116-2), H-RAS (Uniprot: P01112-1), and N-RAS (Uniprot: P01111-1). Degrees of shading according to percentage sequence identity between the four proteins. Asterisk denotes frequently mutated G12 position.

(B) A549 WT or K-RAS$^{GFP/GFP}$ knockin (KI; hereafter called A549^{GFPKRAS}) cell lysates were separated by SDS-PAGE and the indicated antibodies were used for detection by western blotting. Arrows indicate different RAS species (black, unmodified K-RAS; dark gray, GFP-K-RAS; light gray, H-/N-RAS).

(C) Lysates were processed as in (B) and subjected to immunoprecipitation with GFP-trap beads. I, input; Ft, flowthrough; E, elution.

(D) Wide-field immunofluorescence microscopy of untreated A549^{GFPKRAS} cells labeled with antibodies specific for GFP (all left panels, cyan) and P120 (top two middle panels, magenta) or ATPB (bottom two middle panels, magenta), and DAPI (all left and middle panels, blue). Overlay of GFP and P120/ATPB is shown on the right. Scale bars, 10 μm. Two representative images for each staining are shown.

(E) A549 WT (○) or A549^{GFPKRAS} (Δ) cells were treated with cycloheximide (100 μg/mL) and harvested at the indicated time points. Cell lysates were further processed as in (B). Intensities of bands corresponding to K-RAS or GFP-K-RAS were quantified and normalized to GAPDH. Individual values of three experiments are plotted together with the curve of the average of those experiments, relative to the corresponding t0 value. All blots are representative of at least three independent experiments.

that fusion of VHL to an aGFP16 nanobody recruits GFP-tagged proteins, such as VPS34 and PAWS1, to the CUL2-RBX1 E3 ligase machinery for target ubiquitination and subsequent proteasomal degradation (Fulcher et al., 2016). Therefore, we postulated that GFP-K-RAS could be recruited in a similar manner to the CUL2-RBX complex for ubiquitination and degradation (Figure 2A). Indeed, expression of VHL-aGFP16 AdPROM resulted in near complete clearance of GFP-K-RAS from A549^{GFPKRAS} cells compared with the untransduced controls, while the low-molecular-weight band corresponding to H- and/or N-RAS was unaffected (Figure 2B). In contrast, neither VHL nor the aGFP16 nanobody alone, serving as controls, caused any apparent changes in the steady-state levels of GFP-K-RAS or other RAS proteins (Figure 2B). Treatment of VHL-aGFP16 AdPROM expressing A549^{GFPKRAS} cells with the Cullin neddylation inhibitor MLN4924 partially rescued the degradation of GFP-K-RAS compared with DMSO-treated controls (Figure 2C). The neddylation of CUL2 allows a conformational change of the CUL2-RBX E3 ligase machinery so that the RBX E3 ligase can ubiquitinate substrates recruited by VHL. In line with this notion, the levels of HIF1α protein, a bona fide substrate of VHL (Yu et al., 2001), were stabilized upon MLN4924 treatment compared with DMSO control (Figure 2C). Despite the high apparent efficiency of GFP-KRAS degradation by VHL-aGFP16 AdPROM, retroviral transduction of A549^{GFPKRAS} cells often generates uneven levels of AdPROM expression in a mixed population of cells. To get a better understanding of the distribution of the cells within this population, we used a flow cytometric analysis based on GFP fluorescence. We used gates to define a GFP-positive population based on the GFP signal from untransduced A549^{GFPKRAS} cells and using WT A549 cells as a GFP-negative control (Figure 2D). In accordance with the western blot results (Figures 2B), 98% of cells transduced with VHL-aGFP16 AdPROM virus showed GFP-KRAS degradation as compared with untransduced A549^{GFPKRAS} cells (Figure 2D), which manifested in an overall reduction of GFP fluorescence of the single-cell population (Figure 2E). The remaining 2% of A549^{GFPKRAS} cells produced GFP signal comparable with untransduced GFP-positive-population, which could be due to low-level AdPROM expression within these cells (Figure 2D). In contrast, A549^{GFPKRAS} cells expressing VHL or aGFP16 alone were defined as GFP positive at 99.3% or 99.8%, respectively (Figures 2D and 2E).

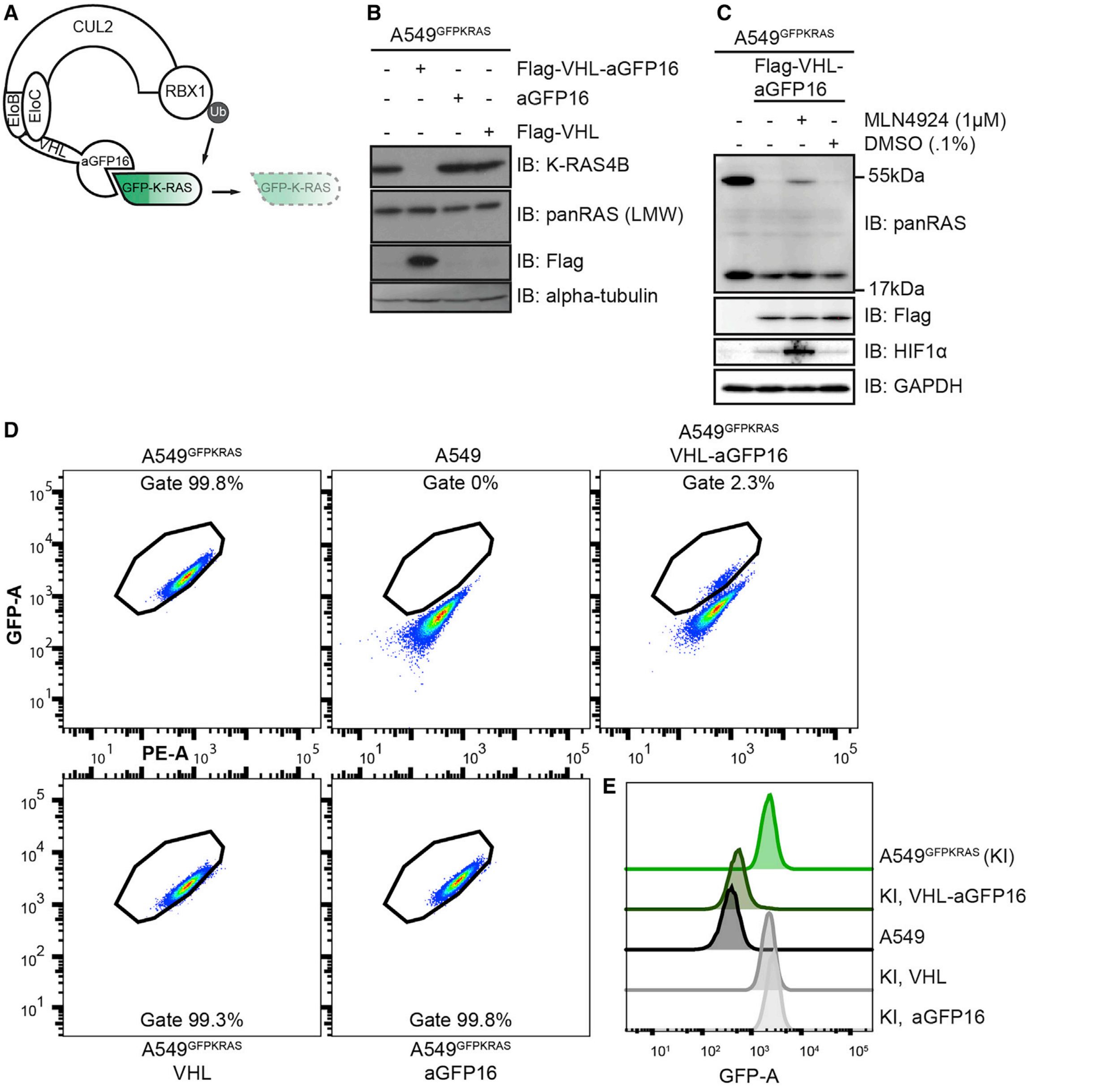

Figure 2. AdPROM-Mediated Degradation of GFP-K-RAS

(A) Schematic representation of the proteolytic AdPROM system. The high-affinity GFP-binder aGFP16 is fused to VHL, which is recruited by EloB and EloC to Cul2. aGFP16 recruits GFP-tagged K-RAS and presents it in close proximity to RBX1 in the assembled Cul2 complex. Ubiquitin (Ub) is transferred onto K-RAS, which is subsequently degraded (dashed lines and faded).

(B) After treatment with retroviruses and selection, cell lysates of indicated cell lines were separated on SDS-PAGE and analyzed by western blotting using the indicated antibodies.

(C) Indicated cell lines were treated with 1 μM MLN4924 in 0.1% DMSO, or just DMSO at 0.1% for 24 h. Samples were further processed as in (B).

(D) Indicated cell lines were analyzed on a Canto flow cytometer. Shown populations were preselected for cells and single cells before defining the gate for GFP-positive cells (shown). GFP-A is plotted against PE-A in all cases. Numbers indicate percentage of cells within the respective gate.

(E) Histogram representation of plots in (D). KI = A549 KRAS$^{GFP/GFP}$ cells (referred to as A549^{GFPKRAS} cells throughout text). Western blots are representative of at least three independent experiments. Flow cytometry data are representative of two independent experiments.

AdPROM-Mediated Degradation of Endogenous RAS Proteins

The AdPROM-mediated degradation of GFP-K-RAS in A549^{GFPKRAS} cells demonstrated the feasibility of targeted degradation of endogenous K-RAS. However, the presence of the GFP tag raises the possibility of ubiquitination occurring on the GFP moiety, instead of K-RAS. Therefore, we sought to explore whether we could exploit the AdPROM system to degrade endogenous, unmodified K-RAS from A549 cells. At present, there are no reported high-affinity, selective

CellPress
OPEN ACCESS

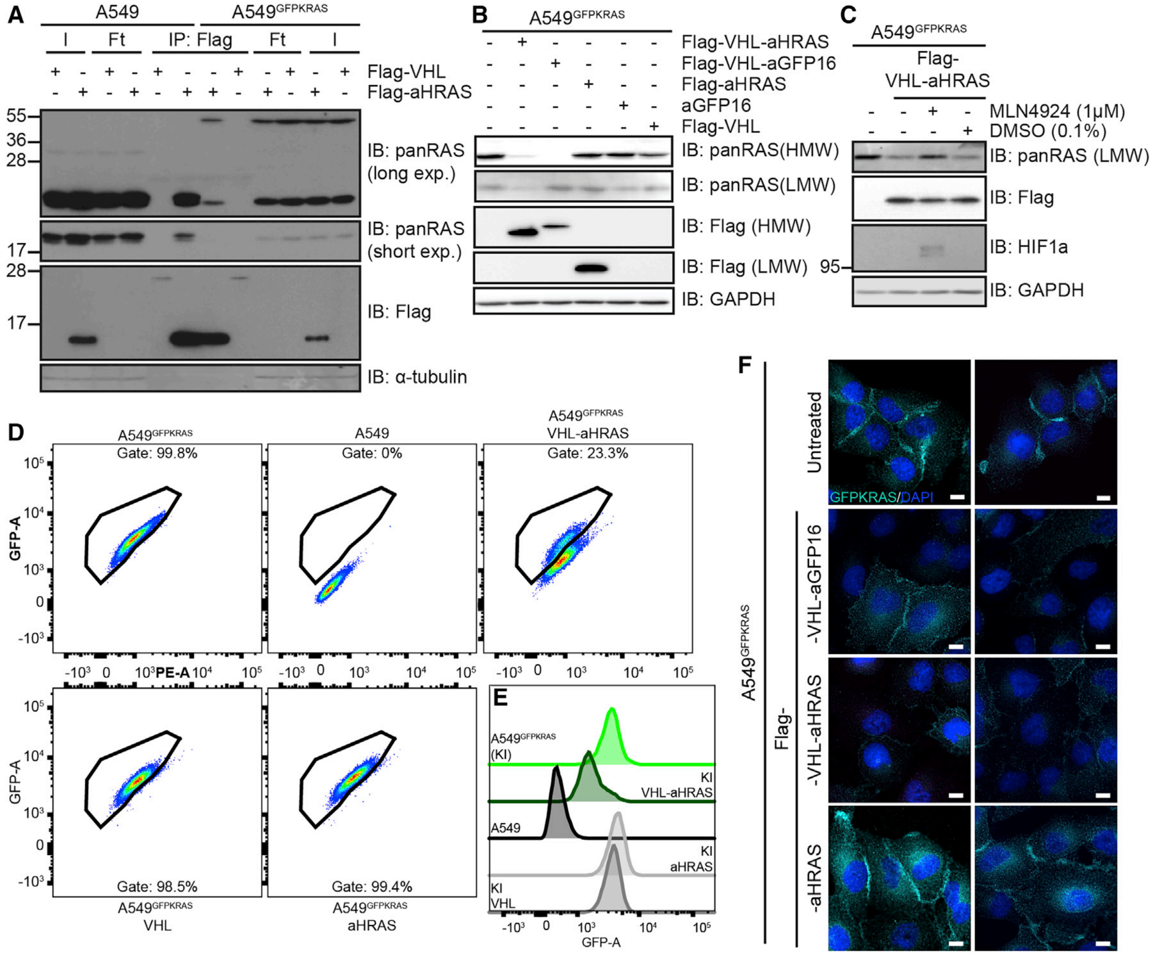

Figure 3. Degradation of Endogenous RAS Using a RAS-Specific Monobody

(A) Cell lysates of indicated cell lines were subjected to immunoprecipitation with anti-Flag beads. Input (I), flowthrough (Ft), and precipitates (IP) were run on SDS-PAGE and subjected to western blotting with the respective antibodies.

(B) After retroviral transduction and selection, cell lysates of indicated cell lines were separated on SDS-PAGE and analyzed by western blotting using the indicated antibodies.

(C) Indicated cell lines were treated with 1 μM MLN4924 or 0.1% DMSO for 24 h. Cell lysates were separated on SDS-PAGE and analyzed by western blotting using the indicated antibodies.

(D) Indicated cell lines were analyzed on a Canto flow cytometer. Shown populations were preselected for cells and single cells before defining the gate for GFP-positive cells (shown). GFP-A is plotted against PE-A in all cases. Numbers indicate percentage of cells within the respective gate.

(E) Histogram representation of plots in (D); KI = A549^{GFPKRAS} cells.

(F) Wide-field immunofluorescence microscopy of indicated cell lines treated with anti-GFP antibody and DAPI for staining. Scale bars, 10 μm. Two representative images are shown for each condition. Western blots and immunofluorescence data are representative of at least three independent experiments. Flow cytometry data are representative of two independent experiments.

polypeptide binders of K-RAS. However, we utilized an anti-H-RAS (aHRAS) monobody that was reported to bind and immunoprecipitate both H-RAS and K-RAS, but not N-RAS (Spencer-Smith et al., 2017). Using this monobody with a FLAG tag, we showed that anti-FLAG immunoprecipitates (IPs) could robustly co-precipitate both GFP-tagged and untagged K-RAS as well as the lower-molecular-weight protein band matching both H- and N-RAS, which is most likely to be H-RAS (Spencer-Smith et al., 2017) (Figure 3A). However, neither of the RAS proteins was completely depleted from flowthrough extracts, suggesting incomplete immunoprecipitation (Figure 3A). In contrast, anti-FLAG IPs from extracts expressing Flag-VHL control did not co-precipitate either protein (Figure 3A).

Next, we sought to investigate whether AdPROM consisting of VHL fused to aHRAS monobody could target K- and H-RAS proteins for degradation. In A549^{GFPKRAS} cells the expression of VHL-aHRAS resulted in a strong reduction of the GFP-K-RAS protein levels when compared with non-transduced, VHL or monobody alone controls (Figure 3B). The degradation induced by VHL-aHRAS AdPROM was slightly less efficient than that achieved with the VHL-aGFP16 AdPROM (Figure 3B). However, unlike VHL-aGFP16, VHL-aHRAS also reduced the protein levels corresponding to the H-RAS and/or N-RAS band (Figure 3B). The loss in protein levels of endogenous H-RAS protein caused by VHL-aHRAS AdPROM could be rescued by the Cullin neddylation inhibitor MLN4924, suggesting that the degradation was mediated through

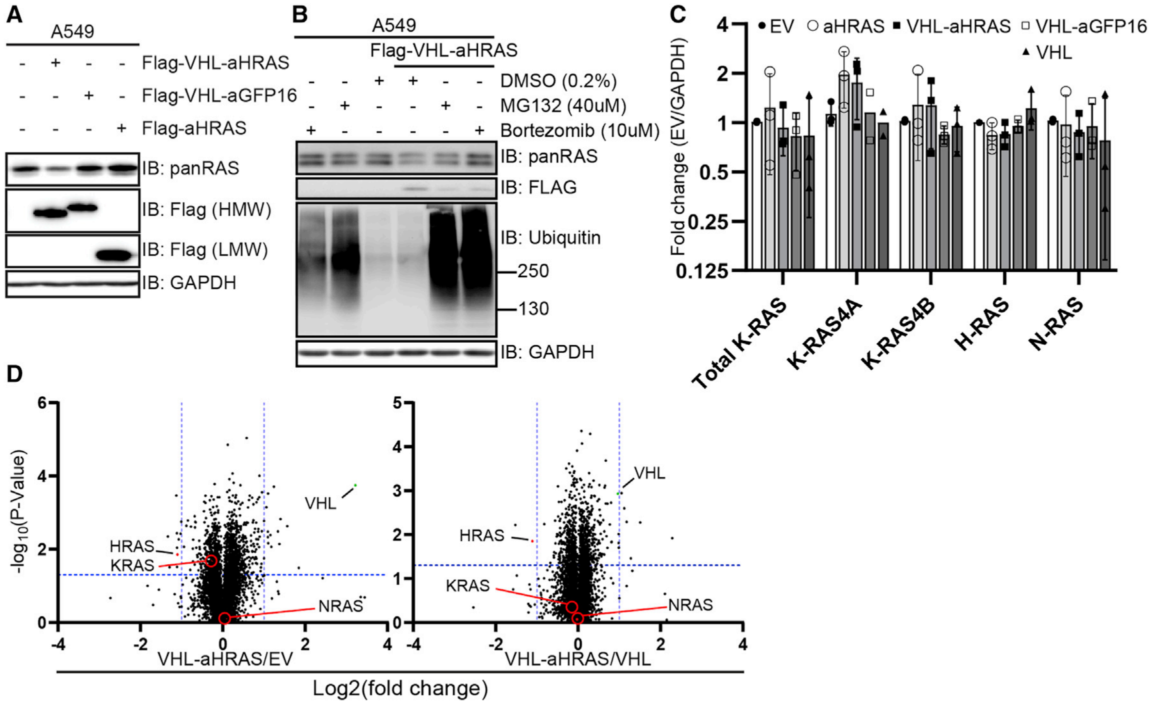

Figure 4. Degradation of Endogenous Unmodified RAS Using a RAS-Specific Monobody

(A) SDS-PAGE and western blot analysis of A549 cells transduced with indicated plasmids with indicated antibodies.

(B) Post-puromycin selection, transduced cells, or non-selected A549 WT cells, were treated with 40 μM MG132, 10 μM bortezomib, or DMSO (all at 0.2% DMSO) for 14 h before harvest.

(C) RNA from A549 cells transduced with and selected for the indicated plasmids was reverse transcribed and screened for mRNA levels by qRT-PCR for the indicated genes, normalized to GAPDH. Error bars (SD) are shown for n = 3 (except K-RAS4A VHL-aGFP16 and VHL, n = 2). Unpaired ordinary one-way ANOVA with Dunnett's multiple comparisons test has been performed.

(D) Volcano plots of proteins identified in tandem mass tag total proteome analysis in A549 cells transduced with and selected for VHL-aHRAS compared with empty vector control (EV) (left) or VHL alone (right). Horizontal line shows significance level of p = 0.05. Vertical lines show 2-fold change. Positions of KRAS, HRAS, NRAS, and VHL are indicated.

CUL2-RBX E3 ligase machinery (Figure 3C). As expected, MLN4924 also stabilized endogenous HIF1α (Figure 3C).

We also assessed the relative abundance of GFP-K-RAS in mixed populations of A549^{GFPKRAS} cells transduced with VHL-aHRAS AdPROM in comparison with controls by flow cytometry. We found that 77% of cells showed degradation of GFP-K-RAS, as assessed by the shift of the GFP-positive gated population toward the GFP-negative population (Figure 3D) and the overall reduction of GFP signal (Figure 3E). The remaining 23% of cells transduced with VHL-aHRAS were seemingly unaffected in terms of GFP level (Figures 3D and 3E). Transductions with VHL or aHRAS alone did not induce any noticeable shift of the GFP population or GFP signal intensity (Figures 3D and 3E).

Uneven retroviral transduction of cells could result in unequal expression of the AdPROM constructs in different cells resulting in a mixed, divergent cell population, which may account for the apparent uneven degradation of GFP-K-RAS through VHL-aHRAS. When we analyzed these A549^{GFPKRAS} mixed cell populations by immunofluorescence for GFP signal, in non-transduced and aHRAS-transduced control cells, a predominant plasma membrane GFP-K-RAS signal was evident (Figure 3F). Transduction of A549^{GFPKRAS} cells with either VHL-aHRAS or VHL-aGFP16 AdPROM produced a heterogeneous population comprising cells with missing or severely attenuated GFP signal, and cells with intact GFP-K-RAS staining pattern, localizing mainly to the plasma membrane (Figure 3F). In contrast, we noticed a slight increase in endoplasmic reticulum (ER)/perinuclear GFP-K-RAS signal in cells transduced with the aHRAS monobody alone (Figure 3F). Interestingly, we detected that the majority of the monobody itself was in the nucleus (Figure S5), while we were unable to consistently detect signals for the AdPROM fusion proteins by anti-FLAG immunofluorescence (Figure S5).

AdPROM-Mediated Degradation of Untagged Endogenous RAS Proteins

Having verified that VHL-aHRAS AdPROM recognizes and degrades GFP-K-RAS, we next tested its ability to degrade endogenous K- and H-/N-RAS in WT A549 cells. The transduction of cells with VHL-aHRAS resulted in a substantial reduction in apparent levels of both K-RAS (upper band) and H-/N-RAS (lower band) proteins as detected by the panRAS antibody and compared with the non-transduced controls (Figure 4A). Unlike in A549^{GFPKRAS} cells (Figure 3B), WT cells transduced with VHL-aGFP16 AdPROM did not display any noticeable changes in K-RAS and H-/N-RAS protein levels relative to

Table 1. Proteins Identified in Total Proteome Analysis in Comparison of VHL-aHRAS versus VHL Transduced Cells, as Either 2-Fold More or Less Abundant

			VHL-aHRAS/VHL		VHL-aHRAS/EV		VHL/EV	
Classification	Protein	Uniprot ID	Fc	p Value	Fc	p Value	Fc	p Value
VHL-aHRAS >2-fold decrease	Neuronal pentraxin-1 (NPTX1)	Q15818	0.348	0.00607	0.961	0.77092	3.028	0.01113
	H-RAS	P01112	0.464	0.01404	0.466	0.01391	0.998	0.84955
VHL-aHRAS >2-fold increase	TFIID subunit 4B (TAF4B)	Q92750	4.875	0.012	3.598	0.03235	0.801	0.25714
	LBH	Q53QV2	2.847	0.00527	1.942	0.08367	0.682	0.38491
	Annexin A8-like protein 1 (ANXA8L1)	Q5VT79	2.434	0.03474	1.487	0.20081	0.511	0.68722
	Enhancer of filamentation 1 (NEDD9)	Q14511	2.268	0.00515	1.508	0.01131	0.72	0.04447
	Transgelin (TAGLN)	Q01995	2.176	0.00251	1.823	0.00281	0.838	0.26401
	M-RAS	O14807	2.079	0.00114	2.017	0.05143	1.013	0.48323
K/N-RAS	K-RAS	P01116	0.909	0.48915	0.841	0.01985	0.886	0.59775
	N-RAS	P01111	0.984	0.84292	1.082	0.82715	1.036	0.86126
VHL and targets	VHL	P40337	1.952	0.00117	9.313	0.00018	4.686	0.00104
	MYBBP1A	Q9BQG0	0.988	0.73087	0.886	0.47828	0.857	0.60345
	RPB1	P24928	1.028	0.78989	0.991	0.75666	0.987	0.24496

Values of identified proteins are given for VHL-aHRAS versus empty vector (EV) and VHL versus EV-transduced cells as well. UniProt ID is given, as well as fold change (F_c) values and p values for the respective comparison. In addition, results are shown for K- and N-RAS, as well as VHL and two described VHL substrates.

non-transduced cells (Figure 4A), further validating the targeted nature of RAS degradation by AdPROM. Cells transduced with the aHRAS monobody alone showed a slight increase in abundance of both K-RAS and H-/N-RAS proteins compared with non-transduced controls (Figure 4A). To ascertain whether AdPROM-mediated degradation occurs via the proteasome, we treated A549 cells expressing the VHL-aHRAS AdPROM system or A549 WT cells with proteasomal inhibitors MG132 and bortezomib, both of which resulted in a strong accumulation of polyubiquitinated proteins (Figure 4B). In A549 WT cells, RAS protein levels increased only slightly after 14 h of MG132 and bortezomib treatment. In contrast, in VHL-aHRAS AdPROM cells, both bortezomib and MG132 rescued RAS protein levels, with bortezomib rescuing it to levels comparable with A549 WT cells (Figure 4B). Next, we sought to explore whether RAS protein degradation triggers a change in RAS transcript levels. We transduced A549 WT cells with constructs encoding Flag-VHL-aHRAS, Flag-aHRAS, Flag-VHL-aGFP16, or Flag-VHL and a pBabeD empty construct as a calibrator. While we noticed a slight increase in K-RAS4A transcripts in cells expressing aHRAS or VHL-aHRAS relative to other cells, these changes were not statistically significant (Figure 4C).

Next, we looked at the global quantitative proteomic changes upon targeted degradation of RAS proteins through the AdPROM system. To this end, we used puromycin-selected cells transduced with pBabeD empty vector, a plasmid encoding Flag-VHL-aHRAS, or a plasmid encoding for Flag-VHL, and performed tandem mass tag-labelled total proteome analysis (Figures 4D and S6). We found that H-RAS was significantly reduced by more than 50% in VHL-aHRAS samples, compared with both VHL alone and the empty vector controls (Figure 4D, Table 1). K-RAS was significantly reduced by ~15% in VHL-aHRAS samples compared with empty vector; however, the reduction was not significant when compared with VHL alone control. N-RAS was unchanged in all conditions, further consolidating the specificity of the monobody toward the H- and K-RAS isoforms (Spencer-Smith et al., 2017). Interestingly, in VHL-aHRAS samples the only proteins significantly downregulated by a factor higher than 2 when compared with VHL alone were H-RAS and NPTX1, the latter, however, most likely stemmed from upregulation by VHL, as the increase was observed in the “VHL alone” sample and it was not changed when compared with the empty vector (Table 1). To our surprise, M-RAS protein abundance increased by 2-fold in cells expressing VHL-aHRAS. In addition, transcription initiation factor TFIID subunit 4B (TAF4B) was more than 3-fold more abundant in the VHL-aHRAS-expressing cells compared with controls (Table 1). The abundance of transcription factor LBH, Annexin-A8-like protein 1 (ANXA8L1), NEDD9, and Transgelin also increased >1.5-fold in VHL-aHRAS-expressing cells compared with controls (Table 1). VHL abundance was roughly 9-fold higher in the VHL-aHRAS-transduced samples compared with the empty vector control and 2-fold higher compared with VHL alone samples (Table 1). While we could not detect HIF1α, we found that overexpression of VHL, either alone or when fused to the aHRAS monobody, did not significantly change protein levels of other VHL substrates (Zhang and Yang, 2012) MYBBP1A (Lai et al., 2011) or RNA polymerase II subunit RPB1 (Kuznetsova et al., 2003).

Expansion of the RAS-Targeting AdPROM System in Different Cell Lines

Having demonstrated for the first time that the VHL-aHRAS AdPROM system could target endogenous, unmodified H- and K-RAS for degradation in A549 cells, we sought to explore whether the system would work in other cell lines. First, we compared different cell lines for their endogenous RAS protein expression (Figure 5A) relative to A549 cells. All cells tested displayed K-RAS protein expression similar to, or slightly lower than, A549

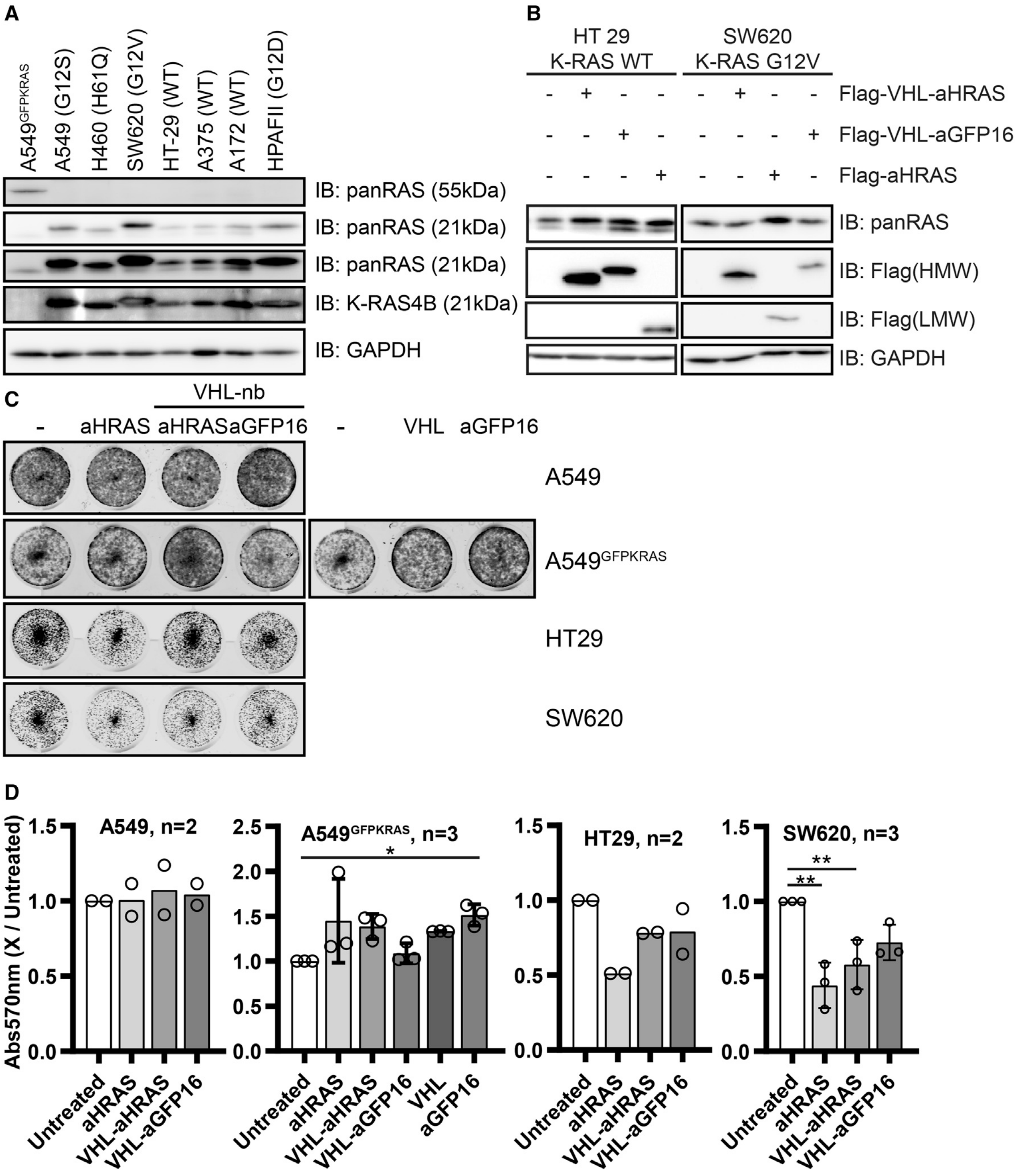

Figure 5. Degradation of RAS in Different Cell Lines and Effects on Proliferation

(A–C) Lysates of untreated (A) or retrovirally transduced cell lines (indicated expression constructs) (B) were separated by SDS-PAGE and analyzed by western blotting with the indicated antibodies. Comparison of cell lines in (A) was done only once. K-RAS mutation statuses for individual cell lines are indicated in brackets. (C) A total of 5,000 cells from (B) or A549 cells from (Figure 4A) or (Figure 3B) were grown in triplicate in 12-well dishes. After 7 days, cells were fixed and stained with crystal violet. A representative image of the replicates is shown.

(D) Staining from plates in (C) was extracted by methanol and absorbance at 570 nm was measured. Plotted 570-nm values are relative to the respective untreated sample. The number of biological replicates (applies to western blots in (B) as well) is indicated next to the cell line and error bars (SD) are shown. For statistical analysis one-way ANOVA analysis with Dunnett's multiple comparisons test was done. Comparisons were drawn to the untreated sample. $^{*}p < 0.05$; $^{**}p < 0.01$.

cells. SW620 cells, which harbor the G12V mutation on K-RAS (Morandi et al., 2012), displayed similar levels of expression to A549 cells; however, we noticed that K-RAS in this cell line produced a slight but noticeable molecular weight shift when probed with panRAS and K-RAS4B antibodies (Figure 5A). Protein levels corresponding to the lower H- and/or N-RAS band were similar in all lines tested but overall, much lower in intensity than that seen for K-RAS. We tested the ability of VHL-aHRAS

CellPress
OPEN ACCESS

AdPROM to degrade RAS proteins from HT-29 and SW620 cells. In HT-29 cells, which express WT RAS proteins but harbor the activating BRAF V600E mutation (Tan et al., 2008), only the levels of H-RAS but not K-RAS proteins were reduced by VHL-aHRAS AdPROM compared with controls (Figure 5B, left panel). For SW620 cells, which harbor the G12V mutation of K-RAS, we noticed a high K-RAS signal to H-/N-RAS signal ratio, as the latter was barely detectable (Figure 5B, right panel). We observed stabilization of K-RAS with the aHRAS monobody alone, while VHL-aHRAS failed to degrade K-RAS compared with controls.

Finally, we wanted to explore whether targeted degradation of K- and H-RAS proteins from WT A549, HT29, and SW620 cells using the VHL-aHRAS AdPROM, and GFP-K-RAS from A549^{GFPKRAS} cells using the VHL-aGFP16 AdPROM would impact cell proliferation. No significant differences in proliferation could be observed for either WT A549 or A549^{GFPKRAS} cells following AdPROM-mediated degradation of the respective RAS proteins compared with controls after 7 days, as measured by crystal violet staining (Figures 5C and 5D). Although A549 cells harbor the oncogenic KRAS^{G12S} mutation, they also harbor over 250 genetic mutations (COSMIC cell lines project) (Tate et al., 2018), including some known oncogenes and tumor suppressors reducing the likelihood that these cells are solely dependent on the K-RAS^{G12S} oncogene for their proliferation. Interestingly, proliferation of HT-29 cells was reduced by about 50% by the aHRAS monobody alone (Figures 5C and 5D), while the VHL-aHRAS and VHL-aGFP16 constructs reduced growth to a lesser extent (Figure 5D). Both the aHRAS monobody alone and the VHL-aHRAS AdPROM, but not VHL-aGFP16 AdPROM were able to reduce the proliferation of SW620 cells significantly by about 50% (Figures 5C and 5D).

DISCUSSION

Overexpression of GFP-tagged or other epitope-tagged K-RAS has been used frequently to investigate RAS localization (Schmick et al., 2014; Spencer-Smith et al., 2017; Tsai et al., 2015). This overcomes the difficulty in the study of RAS proteins in the absence of robust reagents to reliably detect specific RAS proteins at the endogenous levels, especially by immunofluorescence (Waters et al., 2017). Our homozygous A549^{GFPKRAS} NSCLC cell line, generated using CRISPR/Cas9, has allowed us not only to assess localization of endogenously driven GFP-K-RAS protein, but its mobility shift has allowed us to test the utility of panRAS and K-RAS antibodies in detecting K-RAS by western blotting. However, our results also prompt cautious use of GFP-tagged K-RAS, as A549^{GFPKRAS} exhibit a drastically altered phosphorylation status for downstream targets MEK1/2, ERK1/2, and AKT, as well as EGFR. This, of course, might be related to clonal variation; however, in screening for GFP-positive cells following CRISPR-mediated GFP-K-RAS knockin, we only obtained one viable clone, perhaps hinting at a low tolerance for the presence of GFP on endogenous K-RAS. Beyond the plasma membrane localization, we observed additional disperse cytoplasmic signals of endogenous GFP-K-RAS, but no mitochondrial localization. When overexpressed, K-RAS^{G12V} has been suggested to be transported into mitochondria, leading to alterations of membrane potential, a decrease in respiration, and an increase in glycolysis (Hu et al., 2012). Potential compartments for the observed cytosolic signal for K-RAS could be Golgi, as seen for H- and N-RAS (Goodwin et al., 2005), which could correspond to K-RAS4A signal, or ER. However, this remains to be verified.

In this report, we demonstrate that endogenous K-RAS and H-RAS proteins can be targeted for degradation using the proteolytic AdPROM system. RAS proteins have remained elusive targets for anti-cancer therapies, primarily due to their undruggability (Cox et al., 2014). Research into obtaining small-molecule inhibitors of K-RAS has been carried out for over 30 years without much success (Cox and Der, 2010). Recently, RAS-targeting small molecules have emerged, with specificities to (1) a specific mutation status of K-RAS (G12C), i.e., ARS-1620 (Janes et al., 2018), and ARS-853 (Patricelli et al., 2016); (2) K-RAS, independent of the mutation status (McCarthy et al., 2019); or (3) RAS proteins in either nucleotide bound state (Kessler et al., 2019). Two compounds targeting K-RAS^{G12C} mutation, AMG510 and MRTX849, are currently undergoing clinical trials (Lindsay and Blackhall, 2019). An alternative approach has been the development of high-affinity polypeptide binders of RAS that neutralize the RAS function. A class of binders based on ankyrin repeat proteins (Guillard et al., 2017) can bind and neutralize specific nucleotide loading states of RAS proteins (Guillard et al., 2017). Similarly, a fibronectin type III domain-based RAS binding monobody (Khan et al., 2019b; Koide et al., 1998; Spencer-Smith et al., 2017, 2019) was shown to bind and inhibit the dimerization of both K- and H-RAS, and the overexpression of this monobody was shown to suppress tumor growth in mice (Khan et al., 2019b). Besides inhibition, RAS degradation offers another alternative approach at inhibiting RAS function to target RAS-dependent cancer cells. In this context, the dTAG-13 proteolysis targeting chimera (PROTAC) was used to degrade FKBP12^{F36V}-tagged K-RAS (Nabet et al., 2018) through the UPS, albeit when overexpressed in cells. Our AdPROM system demonstrates that endogenous RAS proteins can be targeted for proteolysis through the UPS and suggests that pharmacological targeting of RAS proteins for proteasomal degradation is a viable option for intervention. Although targeted delivery of polypeptide binders of RAS proteins or the proteolytic AdPROM system into RAS-dependent cancer cells remains challenging and currently offers limited therapeutic potential, these are excellent tools to provide the proof of concept. Further optimization of efficient AdPROM gene or protein delivery technologies might enable the study of short-term responses in downstream signaling of RAS. In the clinic and for a thorough analysis of degradation kinetics, cell-permeable small-molecule PROTACs are more viable options than the current AdPROM system, as it relies on long antibiotic selection for transduced cells and in its current form is not tractable. Recently two allosteric small-molecule binders were described for K-RAS with micromolar and nanomolar binding affinities (Kessler et al., 2019; McCarthy et al., 2019). It would be important to test these binders' capabilities as K-RAS targeting warheads in a PROTAC approach, similar to the recently published ones (Bond et al., 2020; Zeng et al., 2020). In this context, a re-evaluation of RAS binding molecules, with or without inhibitory function, might prove successful for PROTAC design. Work published while this study was under review utilizing a KRAS^{G12C}-specific PROTAC harboring a CRBN recruiting warhead was able to

demonstrate the degradation of GFP-K-RAS, but the PROTAC was unable to degrade endogenous or untagged K-RAS (Zeng et al., 2020). Based on these results, this study suggested that targeting endogenous K-RAS for degradation by the proteasome would be difficult, if not impossible. However, our study here demonstrates that VHL-aHRAS AdPROM clearly targets both endogenous H- and K-RAS proteins for degradation through the proteasome. Moreover, a very recent preprint study has demonstrated that a VHL-recruiting K-RAS^{G12C}-specific PROTAC is indeed able to degrade endogenous K-RAS^{G12C} in different cell lines, although its impact on the viability of different cell lines after PROTAC treatment over the K-RAS^{G12C} inhibitors is yet to be addressed (Bond et al., 2020). It has been shown that cells that undergo long-term PROTAC treatment can gain resistance mutations in the E3 ligases or their receptors, which stop the PROTAC-E3 interaction (Zhang et al., 2019). In the search for other E3 ligases applicable for PROTAC development, the AdPROM system represents a rapid research tool with the potential to screen the efficacy of different E3 ligases, or RAS peptide recruiters in degradation of RAS. In addition, our A549^{GFPKRAS} cells provide an excellent high-throughput screening platform to test the efficacy of either new E3 warheads that are compatible with RAS degradation, or new RAS-recruiting warheads.

The field of targeted RAS proteolysis is gaining momentum and presents different approaches for different applications. While the dTAG system offers strong, selective, and more importantly inducible degradation of POIs, it relies on the fusion of FKBP12^{F36V} to the N terminus of RAS (Nabet et al., 2018), which in an endogenous setting might pose problems similar to the effects GFP fusion imposed on K-RAS. In contrast, with the AdPROM system, we were able to degrade endogenous, unmodified RAS proteins, albeit constitutively, which in turn might have allowed cells to adapt to RAS degradation by the time we could perform the analysis of changes in signaling and proliferation. These limitations can be overcome by K-RAS-directed PROTACs which can combine direct degradation of K-RAS with the benefit of small-molecule delivery omitting overexpression of components. However, currently there is a dearth of K-RAS-directed PROTACs, with only two studies reporting the development of K-RAS-G12C-directed PROTACs (Bond et al., 2020; Zeng et al., 2020), while binders for other RAS mutation states are still missing. Nonetheless, in addition to being invaluable research tools, the AdPROM and dTAG systems offer excellent, rapid screening platforms to inform development of PROTACs.

While the VHL-aGFP AdPROM was very effective at selectively degrading GFP-K-RAS from A549^{GFPKRAS} cells, the VHL-aHRAS AdPROM degraded endogenous H- and K-RAS with mixed efficacy in different cell lines. In developing the aHRAS monobody, the authors noted a difference in downstream behaviors of H- and K-RAS upon monobody binding, such as K-RAS, but not H-RAS being displaced from the membrane, or the mutant K-RAS, but not mutant H-RAS interaction with RAF being disturbed by monobody binding (Spencer-Smith et al., 2019). The full determinants of interaction between the aHRAS monobody and different H- and K-RAS mutants or any post-translationally modified forms remain poorly defined. It is perhaps the differences in affinity between the RAS proteins and the aHRAS monobody that define how robustly or poorly VHL-aHRAS can degrade different RAS proteins, as the initially described binding preference of the monobody to H-/K-RAS, but not N-RAS (Spencer-Smith et al., 2017), is reflected in different levels of degradation when used in the AdPROM system (Table 1). Nevertheless, VHL-aHRAS-mediated RAS degradation was enough to elicit a cellular response to the removal of RAS proteins from a cell. Our data indicate a responsive upregulation of both M-RAS and TAF4B in A549 cells. Overexpression of M-RAS with activating mutations has been shown to lead to ERK signaling activation and transformation of cells (Quilliam et al., 1999). TAF4B, on the other hand, is usually associated with oocyte development (Falender et al., 2005a) and spermatogenesis (Falender et al., 2005b), and has not been associated with RAS function before. However, these findings need to be corroborated further.

In our system, it is unclear whether ubiquitination occurs on RAS itself, and/or the monobody, although complex formation would suggest that, within the AdPROM setup, VHL and the monobody are further from the RBX1 ubiquitination zone than bound RAS. In the same line of reasoning, N-terminal GFP of GFP-K-RAS would be even closer to RBX1, which might explain the strong degradation of GFP-K-RAS with VHL-aHRAS. Nonetheless, our study strongly suggests that different high-affinity polypeptide binders that can selectively bind either specific RAS proteins or mutants can be packaged with VHL-AdPROM to target specific RAS proteins for proteasomal degradation. At the same time, endogenous VHL substrates are not affected by the apparent overexpression of VHL (Table 1). We also noted that aHRAS monobody alone resulted in a marked stabilization of both H-RAS and K-RAS in multiple cell lines (Figures 4A and 5B). This effect could be caused either by a feedback loop induced by the inhibition of both RAS species imparted by aH-RAS binding, or by blocking the natural turnover pathway through binding the RAS dimerization interface at helical structures $\alpha4$-$\alpha5$ (Spencer-Smith et al., 2017).

For the cell lines that we used, AdPROM-mediated degradation of H-/K-RAS was not sufficient to induce inhibition of anchorage-dependent cell proliferation. Despite harboring an activating K-RAS^{G12S} mutation, A549 cells do not appear to be strictly dependent on K-RAS alone in anchorage-dependent growth. While A549 cells are often discussed to be K-RAS independent (Kazi et al., 2018; Singh et al., 2009; Symonds et al., 2016), expression of MiR-181a-5p, a microRNA targeting the K-RAS 3′ UTR, reduced A549 anchorage-dependent proliferation and migration. However, MiR-181a-5p does not target K-RAS selectively (Ma et al., 2015). Many RAS-dependent cell proliferation assays use anchorage-independent 3D cultures. For example, the K-RAS^{G12C} drug ARS-1620 was shown to be effective at inhibiting RAS-dependent cell proliferation in 3D cultures but not in 2D cultures (Janes et al., 2018). In a similar manner, EGFR inhibitors show anti-proliferative effects in A549 cells only in an anchorage-independent growth context (Jaramillo et al., 2008). In contrast, SW620 cells, which are considered to be K-RAS dependent (Kazi et al., 2018; Singh et al., 2012), were inhibited in anchorage-dependent proliferation by aHRAS monobody alone. VHL-aHRAS AdPROM, which caused no detectable degradation of K-RAS in these cells, did not inhibit their proliferation any further. The inhibition of cell proliferation of RAS-dependent cells by aHRAS monobody is consistent with previous reports (Khan et al., 2019b; Spencer-Smith et al., 2017). The lack of degradation

CellPress
OPEN ACCESS

of K-RAS by VHL-aHRAS AdPROM could be due to the unusual size shift of K-RAS in these cells, possibly caused by a post-translational modification or a mutation that might allow binding to aH-RAS monobody but prevent ubiquitination by the VHL-AdPROM, although this needs to be defined further. However, considering the length of the transduction and the antibiotic selection process the current AdPROM system uses, at the time of proliferation tests, only cells that have overcome the antiproliferative effects of RAS degradation might be selected. Therefore, to assess the effects of AdPROM-mediated degradation of H-/K-RAS on proliferation robustly, it will be essential to first obtain high-affinity polypeptide RAS binders that bind to specific RAS proteins and then use them in RAS-dependent cell lines using a tractable AdPROM system by either delivering AdPROM proteins or a chemically inducible AdPROM system.

SIGNIFICANCE

Our findings demonstrate clearly that endogenous RAS proteins can be targeted for proteasomal degradation by using the AdPROM system. The system unequivocally informs that targeted proteolysis of endogenous K-RAS is a viable strategy to target K-RAS-dependent pathologies. The findings open up exciting opportunities to develop VHL-recruiting K-RAS-specific cell-permeable PROTACs as potential therapeutic agents. Our findings also highlight the need for developing better and more selective RAS binding polypeptides, such as nanobodies or monobodies, to achieve more selective degradation with the AdPROM system.

STAR★METHODS

Detailed methods are provided in the online version of this paper and include the following:

- KEY RESOURCES TABLE
- RESOURCE AVAILABILITY
  - Lead Contact
  - Materials Availability
  - Data and Code Availability
- EXPERIMENTAL MODEL AND SUBJECT DETAILS
  - Cell Lines
- METHOD DETAILS
  - Sequence Alignment
  - RNA Extraction, cDNA Synthesis and qRT-PCR
  - Cell Line Transfection and Transduction
  - CRISPR/Cas9
  - SDS PAGE and Western Blotting
  - Immunoprecipitation
  - Antibodies
  - Immunofluorescence
  - Cell Proliferation Assays
  - Flow Cytometric Analysis
  - Sample preparation for Tandem Mass Tag (TMT) Labelling
  - LC-MS/MS Mass Spectrometry
  - LC-MS/MS Data Analysis
- QUANTIFICATION AND STATISTICAL ANALYSIS

SUPPLEMENTAL INFORMATION

Supplemental Information can be found online at https://doi.org/10.1016/j.chembiol.2020.06.012.

ACKNOWLEDGMENTS

We thank GS laboratory members for their highly appreciated experimental advice and/or discussions during the course of these experiments. We thank L. Fin, E. Allen, J. Stark, and A. Muir for help and assistance with tissue culture, the staff at the DNA Sequencing services (School of Life Sciences, University of Dundee), and the cloning teams within the MRC-PPU reagents and services (University of Dundee), coordinated by J. Hastie and H. McLauchlan. We thank the staff at the Dundee Imaging Facility (School of Life Sciences, University of Dundee), and the staff at the flow cytometry facility (School of Life Sciences, University of Dundee) for their invaluable help and advice throughout this project.

S.R. is supported by GlaxoSmithKline through the Division of Signal Transduction Therapy collaboration. A.K., K.-H.C., and M.A.Q. are employees of GlaxoSmithKline. G.P.S. is supported by the UK MRC (grant MC_UU_00018/6 and MC_UU_12016/3) and the pharmaceutical companies supporting the Division of Signal Transduction Therapy (Boehringer Ingelheim, GlaxoSmithKline, Merck-Serono).

AUTHOR CONTRIBUTIONS

T.J.M. generated all plasmids used in this study. S.R., A.K., M.A.Q., K.-H.C., and G.P.S. designed the project. S.R. and G.P.S. drafted the manuscript. S.R. acquired and analyzed the data. S.R. and G.P.S. interpreted the data. H.Z. performed mass spectrometric analysis of samples.

DECLARATION OF INTERESTS

M.A.Q., A.K., and K.-H.C. are employees and shareholders of GlaxoSmithKline.

Received: November 8, 2019
Revised: May 12, 2020
Accepted: June 19, 2020
Published: July 14, 2020

REFERENCES

Ahearn, I.M., Haigis, K., Bar-Sagi, D., and Philips, M.R. (2012). Regulating the regulator: post-translational modification of RAS. Nat. Rev. Mol. Cell Biol. *13*, 39–51.

Akutsu, M., Dikic, I., and Bremm, A. (2016). Ubiquitin chain diversity at a glance. J. Cell Sci. *129*, 875–880.

Allan, C., Burel, J.M., Moore, J., Blackburn, C., Linkert, M., Loynton, S., MacDonald, D., Moore, W.J., Neves, C., Patterson, A., et al. (2012). OMERO: flexible, model-driven data management for experimental biology. Nat. Methods *9*, 245–253.

Bond, M.J., Chu, L., Nalawansha, D.A., Li, K., and Crews, C. (2020). Targeted degradation of oncogenic KRASG12C by VHL-recruiting PROTACs. ChemRxiv. https://doi.org/10.26434/chemrxiv.12091176.v1.

Cox, A.D., and Der, C.J. (2010). Ras history. Small GTPases *1*, 2–27.

Cox, A.D., Fesik, S.W., Kimmelman, A.C., Luo, J., and Der, C.J. (2014). Drugging the undruggable RAS: mission possible? Nat. Rev. Drug Discov. *13*, 828–851.

Falender, A.E., Shimada, M., Lo, Y.K., and Richards, J.S. (2005a). TAF4b, a TBP associated factor, is required for oocyte development and function. Dev. Biol. *288*, 405–419.

Falender, A.E., Freiman, R.N., Geles, K.G., Lo, K.C., Hwang, K., Lamb, D.J., Morris, P.L., Tjian, R., and Richards, J.S. (2005b). Maintenance of spermatogenesis requires TAF4b, a gonad-specific subunit of TFIID. Genes Dev. *19*, 794–803.

Fulcher, L.J., Macartney, T., Bozatzi, P., Hornberger, A., Rojas-Fernandez, A., and Sapkota, G.P. (2016). An affinity-directed protein missile system for targeted proteolysis. Open Biol. *6*, 160255.

Fulcher, L.J., Hutchinson, L.D., Macartney, T.J., Turnbull, C., and Sapkota, G.P. (2017). Targeting endogenous proteins for degradation through the affinity-directed protein missile system. Open Biol. *7*, 170066.

Fulcher, L.J., He, Z., Mei, L., Macartney, T.J., Wood, N.T., Prescott, A.R., Whigham, A.J., Varghese, J., Gourlay, R., Ball, G., et al. (2019). FAM83D directs protein kinase CK1α to the mitotic spindle for proper spindle positioning. EMBO Rep. *20*, e47495.

Goodwin, J.S., Drake, K.R., Rogers, C., Wright, L., Lippincott-Schwartz, J., Philips, M.R., and Kenworthy, A.K. (2005). Depalmitoylated Ras traffics to and from the Golgi complex via a nonvesicular pathway. J. Cell Biol. *170*, 261–272.

Guillard, S., Kolasinska-Zwierz, P., Debreczeni, J., Breed, J., Zhang, J., Bery, N., Marwood, R., Tart, J., Overman, R., Stocki, P., et al. (2017). Structural and functional characterization of a DARPin which inhibits Ras nucleotide exchange. Nat. Commun. *8*, 16111.

Hobbs, G.A., Der, C.J., and Rossman, K.L. (2016). RAS isoforms and mutations in cancer at a glance. J. Cell Sci. *129*, 1287–1292.

Hu, Y., Lu, W., Chen, G., Wang, P., Chen, Z., Zhou, Y., Ogasawara, M., Trachootham, D., Feng, L., Pelicano, H., et al. (2012). K-ras G12V transformation leads to mitochondrial dysfunction and a metabolic switch from oxidative phosphorylation to glycolysis. Cell Res. *22*, 399–412.

Janes, M.R., Zhang, J., Li, L.-S., Hansen, R., Peters, U., Guo, X., Chen, Y., Babbar, A., Firdaus, S.J., Darjania, L., et al. (2018). Targeting KRAS mutant cancers with a covalent G12C-specific inhibitor. Cell *172*, 578–589.

Jaramillo, M.L., Banville, M., Collins, C., Paul-Roc, B., Bourget, L., and O'Connor-McCourt, M. (2008). Differential sensitivity of A549 non-small cell lung carcinoma cell responses to epidermal growth factor receptor pathway inhibitors. Cancer Biol. Ther. *7*, 557–568.

John, J., Sohmen, R., Feuerstein, J., Linke, R., Wittinghofer, A., and Goody, R.S. (1990). Kinetics of interaction of nucleotides with nucleotide-free H-Ras p21. Biochemistry *29*, 6058–6065.

Kang, S., Kim, E.-S., and Moon, A. (2009). Simvastatin and lovastatin inhibit breast cell invasion induced by H-Ras. Oncol. Rep. *21*, 1317–1322.

Kazi, A., Xiang, S., Yang, H., Delitto, D., Trevino, J., Jiang, R.H.Y., Ayaz, M., Lawrence, H.R., Kennedy, P., and Sebti, S.M. (2018). GSK3 suppression upregulates β-catenin and c-Myc to abrogate KRas-dependent tumors. Nat. Commun. *9*, 1–9.

Kessler, D., Gmachl, M., Mantoulidis, A., Martin, L.J., Zoephel, A., Mayer, M., Gollner, A., Covini, D., Fischer, S., Gerstberger, T., et al. (2019). Drugging an undruggable pocket on KRAS. Proc. Natl. Acad. Sci. U S A *116*, 15823–15829.

Khan, A.Q., Kuttikrishnan, S., Siveen, K.S., Prabhu, K.S., Shanmugakonar, M., Al- Naemi, H.A., Haris, M., Dermime, S., and Uddin, S. (2019a). RAS-mediated oncogenic signaling pathways in human malignancies. Semin. Cancer Biol. *54*, 1–13.

Khan, I., Spencer-Smith, R., and O'Bryan, J.P. (2019b). Targeting the α4-α5 dimerization interface of K-RAS inhibits tumor formation in vivo. Oncogene *38*, 2984–2993.

Koide, A., Bailey, C.W., Huang, X., and Koide, S. (1998). The fibronectin type III domain as a scaffold for novel binding proteins. J. Mol. Biol. *284*, 1141–1151.

Komander, D., and Rape, M. (2012). The ubiquitin code. Annu. Rev. Biochem. *81*, 203–229.

Kuznetsova, A.V., Meller, J., Schnell, P.O., Nash, J.A., Ignacak, M.L., Sanchez, Y., Conaway, J.W., Conaway, R.C., and Czyzyk-Krzeska, M.F. (2003). von Hippel-Lindau protein binds hyperphosphorylated large subunit of RNA polymerase II through a proline hydroxylation motif and targets it for ubiquitination. Proc. Natl. Acad. Sci. U S A *100*, 2706–2711.

Lai, Y., Qiao, M., Song, M., Weintraub, S.T., and Shiio, Y. (2011). Quantitative proteomics identifies the Myb-binding protein p160 as a novel target of the von Hippel-Lindau tumor suppressor. PLoS One *6*, e16975.

Leung, E.L.H., Luo, L.X., Liu, Z.Q., Wong, V.K.W., Lu, L.L., Xie, Y., Zhang, N., Qu, Y.Q., Fan, X.X., Li, Y., et al. (2018). Inhibition of KRAS-dependent lung cancer cell growth by deltarasin: blockage of autophagy increases its cytotoxicity. Cell Death Dis. *9*, 216.

Lindsay, C.R., and Blackhall, F.H. (2019). Direct Ras G12C inhibitors: crossing the Rubicon. Br. J. Cancer *121*, 197–198.

Livak, K.J., and Schmittgen, T.D. (2001). Analysis of relative gene expression data using real-time quantitative PCR and the $2^{-\Delta\Delta CT}$ method. Methods *25*, 402–408.

Ma, Z., Qiu, X., Wang, D., Li, Y., Zhang, B., Yuan, T., Wei, J., Zhao, B., Zhao, X., Lou, J., et al. (2015). MiR-181a-5p inhibits cell proliferation and migration by targeting Kras in non-small cell lung cancer A549 cells. Acta Biochim. Biophys. Sin. *47*, 630–638.

Madeira, F., Park, Y.M., Lee, J., Buso, N., Gur, T., Madhusoodanan, N., Basutkar, P., Tivey, A.R.N., Potter, S.C., Finn, R.D., et al. (2019). The EMBL-EBI search and sequence analysis tools APIs in 2019. Nucleic Acids Res. *47*, W636–W641.

Marcus, K., and Mattos, C. (2015). Direct attack on RAS: intramolecular communication and mutation-specific effects. Clin. Cancer Res. *21*, 1810–1818.

McCarthy, M.J., Pagba, C.V., Prakash, P., Naji, A.K., van der Hoeven, D., Liang, H., Gupta, A.K., Zhou, Y., Cho, K.-J., Hancock, J.F., et al. (2019). Discovery of high-affinity noncovalent allosteric KRAS inhibitors that disrupt effector binding. ACS Omega *4*, 2921–2930.

Morandi, L., de Biase, D., Visani, M., Cesari, V., De Maglio, G., Pizzolitto, S., Pession, A., and Tallini, G. (2012). Allele specific locked nucleic acid quantitative PCR (ASLNAqPCR): an accurate and cost-effective assay to diagnose and quantify KRAS and BRAF mutation. PLoS One *7*, e36084.

Nabet, B., Roberts, J.M., Buckley, D.L., Paulk, J., Dastjerdi, S., Yang, A., Leggett, A.L., Erb, M.A., Lawlor, M.A., Souza, A., et al. (2018). The dTAG system for immediate and target-specific protein degradation. Nat. Chem. Biol. *14*, 431–441.

O'Bryan, J.P. (2019). Pharmacological targeting of RAS: recent success with direct inhibitors. Pharmacol. Res. *139*, 503–511.

Ostrem, J.M., Peters, U., Sos, M.L., Wells, J.A., and Shokat, K.M. (2013). K-Ras(G12C) inhibitors allosterically control GTP affinity and effector interactions. Nature *503*, 548–551.

Pai, E.F., Kabsch, W., Krengel, U., Holmes, K.C., John, J., and Wittinghofer, A. (1989). Structure of the guanine-nucleotide-binding domain of the Ha-ras oncogene product p21 in the triphosphate conformation. Nature *341*, 209–214.

Papke, B., and Der, C.J. (2017). Drugging RAS: know the enemy. Science *355*, 1158–1163.

Patricelli, M.P., Janes, M.R., Li, L.-S., Hansen, R., Peters, U., Kessler, L.V., Chen, Y., Kucharski, J.M., Feng, J., Ely, T., et al. (2016). Selective inhibition of oncogenic KRAS output with small molecules targeting the inactive state. Cancer Discov. *6*, 316–329.

Pickart, C.M., and Eddins, M.J. (2004). Ubiquitin: structures, functions, mechanisms. Biochim. Biophys. Acta *1695*, 55–72.

Quilliam, L.A., Castro, A.F., Rogers-Graham, K.S., Martin, C.B., Der, C.J., and Bi, C. (1999). M-Ras/R-Ras3, a transforming Ras protein regulated by Sos1, GRF1, and p120 Ras GTPase-activating protein, interacts with the putative Ras effector AF6. J. Biol. Chem. *274*, 23850–23857.

Reiss, Y., Goldstein, J.L., Seabra, M.C., Casey, P.J., and Brown, M.S. (1990). Inhibition of purified $p21^{ras}$ farnesyl:protein transferase by Cys-AAX tetrapeptides. Cell *62*, 81–88.

Reynolds, A.B., Daniel, J., McCrea, P.D., Wheelock, M.J., Wu, J., and Zhang, Z. (1994). Identification of a new catenin: the tyrosine kinase substrate p120cas associates with E-cadherin complexes. Mol. Cell. Biol. *14*, 8333–8342.

Roos-Mattjus, P., and Sistonen, L. (2004). The ubiquitin-proteasome pathway. Ann. Med. *36*, 285–295.

Röth, S., Fulcher, L.J., and Sapkota, G.P. (2019). Advances in targeted degradation of endogenous proteins. Cell. Mol. Life Sci. *76*, 2761–2777.

Schaber, M.D., O'Hara, M.B., Garsky, V.M., Mosser, S.D., Bergstrom, J.D., Moores, S.L., Marshall, M.S., Friedman, P.A., Dixon, R.A.F., and Gibbs, J.B.

CellPress
OPEN ACCESS

(1990). Polyisoprenylation of Ras in vitro by a farnesyl-protein transferase. J. Biol. Chem. *265*, 14701–14704.

Schatz, G., and Butow, R.A. (1983). How are proteins imported into mitochondria? Cell *32*, 316–318.

Schmick, M., Vartak, N., Papke, B., Kovacevic, M., Truxius, D.C., Rossmannek, L., and Bastiaens, P.I.H. (2014). KRas localizes to the plasma membrane by spatial cycles of solubilization, trapping and vesicular transport. Cell *157*, 459–471.

Singh, A., Greninger, P., Rhodes, D., Koopman, L., Violette, S., Bardeesy, N., and Settleman, J. (2009). A gene expression signature associated with "K-Ras addiction" reveals regulators of EMT and tumor cell survival. Cancer Cell *15*, 489–500.

Singh, A., Sweeney, M.F., Yu, M., Burger, A., Greninger, P., Benes, C., Haber, D.A., and Settleman, J. (2012). TAK1 inhibition promotes apoptosis in KRAS-dependent colon cancers. Cell *148*, 639–650.

Spencer-Smith, R., Koide, A., Zhou, Y., Eguchi, R.R., Sha, F., Gajwani, P., Santana, D., Gupta, A., Jacobs, M., Herrero-Garcia, E., et al. (2017). Inhibition of RAS function through targeting an allosteric regulatory site. Nat. Chem. Biol. *13*, 62–68.

Spencer-Smith, R., Li, L., Prasad, S., Koide, A., Koide, S., and O'Bryan, J.P. (2019). Targeting the α4-α5 interface of RAS results in multiple levels of inhibition. Small GTPases *10*, 378–387.

Symonds, J.M., Ohm, A.M., Tan, A.-C., and Reyland, M.E. (2016). PKCδ regulates integrin αVβ3 expression and transformed growth of K-ras dependent lung cancer cells. Oncotarget *7*, 17905–17919.

Tan, Y.H., Liu, Y., Eu, K.W., Ang, P.W., Li, W.Q., Tellez, M.S., Iacopetta, B., and Soong, R. (2008). Detection of BRAF V600E mutation by pyrosequencing. Pathology *40*, 295–298.

Tate, J.G., Bamford, S., Jubb, H.C., Sondka, Z., Beare, D.M., Bindal, N., Boutselakis, H., Cole, C.G., Creatore, C., Dawson, E., et al. (2018). COSMIC: the catalogue of somatic mutations in cancer. Nucleic Acids Res. *47*, 941–947.

The UniProt Consortium (2019). UniProt: a worldwide hub of protein knowledge. Nucleic Acids Res. *47*, D506–D515.

Tovell, H., Testa, A., Maniaci, C., Zhou, H., Prescott, A.R., Macartney, T., Ciulli, A., and Alessi, D.R. (2019). Rapid and reversible knockdown of endogenously tagged endosomal proteins via an optimized HaloPROTAC degrader. ACS Chem. Biol. *14*, 882–892.

Tsai, F.D., Lopes, M.S., Zhou, M., Court, H., Ponce, O., Fiordalisi, J.J., Gierut, J.J., Cox, A.D., Haigis, K.M., and Philips, M.R. (2015). K-Ras4A splice variant is widely expressed in cancer and uses a hybrid membrane-targeting motif. Proc. Natl. Acad. Sci. U S A *112*, 779–784.

Vigil, D., Cherfils, J., Rossman, K.L., and Der, C.J. (2010). Ras superfamily GEFs and GAPs: validated and tractable targets for cancer therapy? Nat. Rev. Cancer *10*, 842–857.

Waldmann, H., Karaguni, I.-M., Carpintero, M., Gourzoulidou, E., Herrmann, C., Brockmann, C., Oschkinat, H., and Müller, O. (2004). Sulindac-derived Ras pathway inhibitors target the Ras-Raf interaction and downstream effectors in the Ras pathway. Angew. Chem. Int. Ed. *43*, 454–458.

Waterhouse, A.M., Procter, J.B., Martin, D.M.A., Clamp, M., and Barton, G.J. (2009). Jalview version 2—a multiple sequence alignment editor and analysis workbench. Bioinformatics *25*, 1189–1191.

Waters, A.M., Ozkan-Dagliyan, I., Vaseva, A.V., Fer, N., Strathern, L.A., Hobbs, G.A., Tessier-Cloutier, B., Gillette, W.K., Bagni, R., Whiteley, G.R., et al. (2017). Evaluation of the selectivity and sensitivity of isoform- and mutation-specific RAS antibodies. Sci. Signal. *10*, eaao3332.

Winter, G.E., Buckley, D.L., Paulk, J., Roberts, J.M., Souza, A., Dhe-Paganon, S., and Bradner, J.E. (2015). Phthalimide conjugation as a strategy for in vivo target protein degradation. Science *348*, 1376–1381.

Yau, R., and Rape, M. (2016). The increasing complexity of the ubiquitin code. Nat. Cell Biol. *18*, 579–586.

Yu, F., White, S.B., Zhao, Q., and Lee, F.S. (2001). HIF-1alpha binding to VHL is regulated by stimulus-sensitive proline hydroxylation. Proc. Natl. Acad. Sci. U S A *98*, 9630–9635.

Zeng, M., Xiong, Y., Safaee, N., Nowak, R.P., Donovan, K.A., Yuan, C.J., Nabet, B., Gero, T.W., Feru, F., Li, L., et al. (2020). Exploring targeted degradation strategy for oncogenic KRASG12C. Cell Chem. Biol. *27*, 19–31.

Zhang, Q., and Yang, H. (2012). The roles of VHL-dependent ubiquitination in signaling and cancer. Front. Oncol. *2*, 1–7.

Zhang, L., Riley-Gillis, B., Vijay, P., and Shen, Y. (2019). Acquired resistance to BET-ProTACS (proteolysis-targeting chimeras) caused by genomic alterations in core components of E3 ligase complexes. Mol. Cancer Ther. *18*, 1302–1311.

STAR★METHODS

KEY RESOURCES TABLE

REAGENT or RESOURCE	SOURCE	IDENTIFIER
Antibodies		
Alpha tubulin Monoclonal antibody (YOL1/34)	Thermo Fisher	Cat# MA1-80189; RRID: AB_2210200
Goat anti-rat IgG Secondary Antibody (HRP)	Thermo Fisher	Cat# 31470; RRID: AB_228356
B-RAF Rabbit Monoclonal Antibody (7H30L21)	Thermo Fisher	Cat# 702187; RRID: AB_2633065
Donkey Anti-Rabbit IgG, AlexaFluor 488)	ThermoFisher	Cat# A21206; RRID: AB_2535792
Goat anti-Mouse IgG, AlexaFluor 594	ThermoFisher	Cat# A11005; RRID: AB_2534073
Anti-KRAS+HRAS+NRAS antibody [EPR18713-13]	Abcam	Cat# Ab206969
Anti-HIF-1 alpha antibody [H1alpha67]	Abcam	Cat# Ab1; RRID: AB_296474
Anti-ATPB antibody [3D5]	Abcam	Cat# Ab14730; RRID: AB_301438
Monoclonal Anti-KRAS antibody	SigmaAldrich	Cat# WH0003845M1; RRID: AB_1842235
Monoclonal Anti-Flag M2-Peroxidase (HRP) antibody	SigmaAldrich	Cat# A8592; RRID: AB_439702
Anti GFP from mouse IgG_{1K} (clones 7.1 and 13.1)	SigmaAldrich	Cat# 11814460001; RRID: AB_390913
Monoclonal Anti-FLAG M2 antibody	SigmaAldrich	Cat# F1804; RRID: AB_262044
GAPDH (12C10) rabbit mAb	CST	Cat# 2118S; RRID: AB_561053
P44/42 MAPK (Erk1/2) Antibody	CST	Cat# 9102S; RRID: AB_330744
Phospho-p44/42 MAPK (Erk1/2) (Thr202/Tyr204) (E10) Mouse mAb	CST	Cat# 9106S; RRID: AB_331768
MEK1/2 (L38C12) Mouse mAb	CST	Cat# 4694S; RRID: AB_10695868
Phospho-MEK1/2 (Ser221) (166F8) Rabbit mAb	CST	Cat# 2338S; RRID: AB_490903
AKT Antibody	CST	Cat# 9272S; RRID: AB_329827
Phospho-Akt (Ser473) (D9W9U) Mouse mAb	CST	Cat# 12694S; RRID: AB_2797994
Phospho-EGF Receptor (Tyr1068) (D7A5) XP Rabbit mAb	CST	Cat# 3777; RRID: AB_2096270
C-Myc (D84C12) Rabbit mAb	CST	Cat# 5605; RRID: AB_1903938
Anti-rabbit IgG, HRP-linked Antibody	CST	Cat# 7074S; RRID: AB_2099233
Anti-mouse IgG, HRP-linked Antibody	CST	Cat# 7076S; RRID: AB_330924
EGFR (1005)-G Antibody	SantaCruz	Cat# sc-03-G; RRID: AB_631420
StarBright Blue 700 Goat Anti-Rabbit IgG	BioRad	Cat# 12004161; RRID: AB_2721073
NRAS Rabbit polyclonal antibody	Proteintech	Cat# 10724-1-AP; RRID: AB_2154209
HRAS Rabbit-Polyclonal Antibody	Proteintech	Cat# 18295-1-AP; RRID: AB_2121046
KRAS-2B Rabbit Polyclonal Antibody	Proteintech	Cat# 16155-1-AP; RRID: AB_2134119
KRAS-2A Rabbit Polyclonal Antibody	Proteintech	Cat# 16156-1-AP; RRID: AB_2234477
H-RAS Polyclonal Antibody	Invitrogen	Cat# PA5-22392; RRID: AB_11152295
K-RAS Monoclonal Antibody (9.13)	Invitrogen	Cat# 415700; RRID: AB_2532192
GFP Polyclonal Antibody	MBL/Caltag	Cat# 598; RRID: AB_591819
Mouse Anti-p120 Catenin Antibody [clone 98/pp120]	BD Biosciences	Cat# 610133; RRID: AB_397536
Chemicals, Peptides, and Recombinant Proteins		
Cycloheximide	SigmaAldrich	Cat# C1988

(*Continued on next page*)

Continued

REAGENT or RESOURCE	SOURCE	IDENTIFIER
Bortezomib	LC Laboratories	Cat# B-1408
MG132	abcam	Cat# Ab141003
PEI MAX – Transfection Grade Linear PEI Hydrochloride MW 40,000	Polysciences	Cat# 24765
Polybrene (Hexadimethrine bromide)	SigmaAldrich	Cat# 107689
GFP-Trap-Agarose	Chromotek	Cat# GTA-20; RRID: AB_2631357
Anti-Flag M2 Affinity Gel	Merck	Cat# A2220; RRID: AB_10063035
Vectashield Antifade mounting medium	Vector Labs	Cat# H-1000; RRID: AB_2336789
Lys-C Protease, MS Grade	Alpha Labs	Cat# 125-05061
Pierce Trypsin Protease, MS Grade	ThermoFisher	Cat# 90058
Immobilon Western Chemiluminescent HRP Substrate	Merck	Cat# WBKLS0500
Critical Commercial Assays		
iScript cDNA synthesis Kit	Biorad	Cat# 1708891
SsoFast EvaGreen Supermix	Biorad	Cat# 1725204
TMT10plex Isobaric Label Reagent Set	ThermoFisher	Cat# 90110
Deposited Data		
Results from TMT9plex labelling and MS analysis, see Table S1-A549-ADPROM-TMT9plex-Related to Figure 4D	This paper	N/A
Data obtained in this study	This paper	osf.io/zm3dx
Experimental Models: Cell Lines		
A549	ATCC	Cat# CCL-185; RRID: CVCL_0023
A549^{GFPKRAS}	This paper	N/A
HEK293-FT	Invitrogen	Cat# R70007
A375	ATCC	Cat# CRL-1619; RRID: CVCL_0132
A172	ATCC	Cat# CRL-1620; RRID: CVCL_0131
HT-29	ATCC	Cat# HTB-38; RRID: CVCL_0320
HPAFII	ATCC	Cat# CRL-1997; RRID: CVCL_0313
H460	ATCC	Cat# HTB-177; RRID: CVCL_0459
SW620	ATCC	Cat# CCL-227; RRID: CVCL_0547
Oligonucleotides		
Primers for qRT-PCR, Screening & Sequencing see Table S2– Primer Sequences – Related to STAR Methods	This paper	N/A
Recombinant DNA		
pBabeD P U6 KRAS Nter KI Sense	This paper; MRCPPU Reagents and Services	Cat# DU54976
pX335 KRAS Nter KI AntiSense	This paper; MRCPPU Reagents and Services	Cat# DU54980
pMK-RQ KRAS G12S Nter GFP donor	This paper; MRCPPU Reagents and Services	Cat# DU57406
pBABED P FLAG aHRAS nanobody	This paper; MRCPPU Reagents and Services	Cat# DU57190
pBABED P FLAG VHL aHRAS nanobody	This paper; MRCPPU Reagents and Services	Cat# DU57191
pBABED P FLAG VHL	Fulcher et al. 2017; MRCPPU Reagents and Services	Cat# DU54477
pBABED P aGFP16	Fulcher et al. 2016, MRCPPU Reagents and Services	Cat# DU54238

(*Continued on next page*)

Continued

REAGENT or RESOURCE	SOURCE	IDENTIFIER
pBABED P FLAG VHL-aGFP.16	This paper; MRCPPU Reagents and Services	Cat# DU54295
pBabeD puro (empty vector)	MRCPPU Reagents and Services	Cat# DU33769
pCMV Gag pol	Cell Biolabs	Cat# RV-111
pCMV VSV-G	Cell Biolabs	Cat# RV-110
Software and Algorithms		
Uniprot	The UniProt Consortium, 2019	https://uniprot.org
Clustal Omega	Madeira et al., 2019	https://ebi.ac.uk/Tools/msa/clustalo/
JalView	Waterhouse et al., 2009)	https://jalview.org
ImageLab	BioRad	N/A
SoftWoRx	GE Healthcare	N/A
OMERO	Allan et al., 2012	http://openmicroscopy.org/
Graphpad Prism	GraphPad Prism Inc	N/A

RESOURCE AVAILABILITY

Lead Contact

Further information and requests for resources and reagents should be directed to and will be fulfilled by the Lead Contact, Gopal Sapkota (G.Sapkota@dundee.ac.uk).

Materials Availability

Plasmids generated in this study can be obtained from MRC PPU Reagents and Services (https://mrcppureagents.dundee.ac.uk/).

Data and Code Availability

Original data have been deposited to the Center for Open Science repository: osf.io/zm3dx

EXPERIMENTAL MODEL AND SUBJECT DETAILS

Cell Lines

$A549^{GFPKRAS}$ cells were derived from the epithelial lung cancer cell line A549 by CRISPR/Cas9 mediated knock-in of GFP CDS to the KRAS locus. A549 cells were derived from a 58 year old Caucasian male. A375 cells are a malignant melanoma cell line from a 54 year old female. A172 cells are glioblastoma cells from a 53 year old male. SW620 cells are Duke's type C colorectal adenocarcinoma cells derived from the lymph node metastatic site of a 51 year old Caucasian male. HT-29 cells are colorectal adenocarcinoma cells derived from a primary tumor from a 44 year old Caucasian female. HPAFII cells are pancreatic adenocarcinoma cells derived from peritoneal ascitic fluid of a 44 year old Caucasian male. H460 cells are large cell lung cancer cells derived from pleural effusion of a male patient. HEK293-FT cells are a clonal isolate of HEK293 human embryonic kidney cells transformed with the SV40 large T antigen. All cells were cultured in humidified incubators at 37°C and 5% CO_2. A549, HEK293-FT, A375, A172 and SW620 cells were cultured in Dulbecco's modified Eagle's medium (DMEM; Gibco) with 10% FBS (Sigma), 1% penicillin/streptomycin (Lonza) and 2 mM L-glutamine (Lonza). HT-29, HPAFII and H460 cells were cultured in RPMI1640 medium (Gibco), with the same supplements as DMEM.

METHOD DETAILS

Sequence Alignment

Protein sequences of K-RAS4A/B, H-RAS and N-RAS were taken from Uniprot (The UniProt Consortium, 2019) and aligned in Clustal Omega (Madeira et al., 2019). The alignment was further processed in JalView (Waterhouse et al., 2009) to highlight percent sequence identity.

RNA Extraction, cDNA Synthesis and qRT-PCR

For RNA extraction, $2x10^5$ cells were seeded in a 6-well dish and harvested the next day with the RNeasy Micro Kit (Qiagen, #74004) according to the manufacturer's protocol. 1 μg of RNA was reverse transcribed with the iScript cDNA synthesis Kit (BIORAD, #1708891) according to the manufacturer's protocol. For qRT-PCR 1 μl of diluted cDNA (1:20 or 1:80) was mixed with forward and reverse primers (Custom primers from Invitrogen, 300 nM final concentration each) and SsoFast EvaGreen Supermix (BIORAD, #1725204) in a 384-well plate (Axygen, #321-22-051) and run on a BIORAD CFX384.

Primer sequences:

K-RAS4A fw: GAGGGAGATCCGACAATACAG;
K-RAS4A rev: TCTCGAACTAATGTATAGAAGGCATC;
K-RAS4Bfw: TTGCCTTCTAGAACAGTAGACAC;
K-RAS4B rev: CATCGTCAACACCCTGTCTTG;
Total K-RAS fw: GGAGTACAGTGCAATGAGGG;
Total K-RAS rev: CCATAGGTACATCTTCAGAGTCC;
H-RAS fw: GAACAAGTGTGACCTGGCT;
H-RAS rev: ACCAACGTGTAGAAGGCATC;
N-RAS fw: AATACATGAGGACAGGCGAAG;
N-RAS rev: GTTTCCCACTAGCACCATAGG;
GAPDH fw: CTTTGTCAAGCTCATTTCCTGG;
GAPDH rev: TCTTCCTCTTGTGCTCTTGC.

Following the PCR, melting curves were generated with default settings between 65°C and 95°C in 0.5°C steps at 5 sec intervals. Melting curves were manually analysed for purity of the PCR product, i.e. consistency of amplicon melting temperature between different samples and peak distribution. Fold changes of transcripts were calculated by the 2-ΔΔCt method (Livak and Schmittgen, 2001).

Cell Line Transfection and Transduction

For retrovirus production, 3.2 μg pCMV-gag-pol (Cell Biolabs, RV-111), 2.2 μg pCMV-VSV-G (Cell Biolabs, RV-110) and 6 μg of respective pBabeD plasmids (Flag-aHRAS, DU57190; Flag-VHL-aHRAS, DU57191; Flag-VHL, DU54477; aGFP16, DU54238; Flag-VHL-aGFP16, DU54295) were co-transfected in roughly 70% confluent HEK293-FT cells cultured on a 10-cm dish. Plasmids were mixed with 600 μl Opti-MEM (Gibco) and 24 μl of 1 mg/ml polyethyleneimine (Polysciences) dissolved in 25 mM HEPES pH 7.5. The mixture was vigorously vortexed for 15 s and incubated for 20 min at room temperature. The volume was adjusted to 10 ml with DMEM and added to FT cells. After 24 h, medium was exchanged to DMEM or RPMI, depending on the target cell growth medium. After an additional 24 h, the medium was harvested and filtered through a 0.45 μm Minisart syringe filter (Sartorius). The supernatant was added to a plate of roughly 70% confluent target cells in a 1:10–1:4 dilution (in respective medium) in the presence of 8 μg/ml polybrene (Sigma). After 24 h, growth medium was exchanged with fresh medium containing 2 μg/ml puromycin, to select transduced cells. Puromycin was removed from the medium after 48 h. For inhibitor experiments cells were treated with cycloheximide (100μg/ml; SigmaAldrich, C1988), MLN4924 (1μM, 24 hours, MRC-PPU Reagents and Services), MG132 (40μM, 14 hours, abcam, ab141003), Bortezomib (10μM, 14 hours, LC Laboratories, B-1408) or DMSO (adjusted to match respective inhibitor; SigmaAldrich, D2650).

Cells were lysed on ice, by washing once with PBS and scraping in lysis buffer (50 mM Tris–HCl pH 7.5, 0.27 M sucrose, 150 mM NaCl, 1 mM EGTA, 1 mM EDTA, 1 mM sodium orthovanadate, 1 mM sodium β-glycerophosphate, 50 mM sodium fluoride, 5 mM sodium pyrophosphate, 1% (v/v) Triton X-100 and 0.5% Nonidet P-40) supplemented with protease inhibitors (Roche; 1 tablet/25 ml of lysis buffer). Protein content from cleared cell lysates was determined with Pierce Detergent Compatible Bradford Assay Kit (Thermo Fisher). Lysates were processed further or frozen and stored at -20°C.

CRISPR/Cas9

For generation of N-terminal GFP knock-in A549 cell lines the KRAS locus was targeted with a dual guide approach (Fulcher et al., 2019) (using the sense guide (pBabeD vector, DU54976): GCGAATATGATCCAACAATAG; antisense guide (pX335 vector, DU54980): GCTGAATTAGCTGTATCGTCA; and the GFP-KRAS donor (pMK-RQ vector, DU57406). Briefly, 1 μg of each of the guideRNA plasmids and 3 μg of the donor plasmid were co-transfected into A549 cells. Plasmids were mixed with 1 ml of Opti-MEM (Gibco) and 20 μl of 1 mg/ml polyethyleneimine (Polysciences), vortexed vigorously for 15 s and added to 70% confluent cells in a 10-cm dish. The next day, cells were selected in puromycin (2.5 μg/ml) for 48 h and re-transfected with the same plasmids once they reached 70% confluence. Single GFP positive cells were obtained by FACS sorting and surviving single cell clones were screened by genomic DNA based PCR and Western blot to validate homozygous knock-in of the GFP-tag on the endogenous *KRAS* gene. For PCR based screening the following primers were used: Fw: ATCCAAGAGAACTACTGCCATGATGC;

Rv: CATGACCTTCAAGGTGTCTTACAGGTC. PCR products of positive clones were cloned with the StrataClone PCR Cloning Kit (Agilent) into the supplied vector system, according to the manufacturer's protocol. Sequencing of positive clones was carried out by the MRC-PPU DNA Sequencing and Services with a custom primer close to the RAS mutation site (Rv: CAAAGAATGGTCCTGCACCAG).

SDS PAGE and Western Blotting

Cell lysates were adjusted to uniform protein concentration and mixed with 6x reducing Laemmli SDS sample buffer (Fisher Scientific). 10-20 μg of total lysate protein, or immunoprecipitates were resolved by SDS polyacrylamide gel electrophoeresis (PAGE). After PAGE, proteins were transferred onto methanol activated PVDF membrane (Immobilon-P or Immobilon-FL, Merck) in Tris/glycine buffer containing 20% methanol in a tank blotting system for 85 min at a constant voltage of 85 V. The membranes were then re-incubated with methanol for 2 minutes and stained with Ponceau S solution to gauge uniform protein transfer (Sigma). After de-staining membranes in TBS-T (50 mM Tris–HCl pH 7.5, 150 mM NaCl, 0.1% Tween-20), they were blocked for 1 h in 5% non-

fat milk (Marvel) in TBS-T. Primary antibody incubation was done overnight at 4°C in 5% milk/TBS-T. Following 3x10 min washes in TBS-T, membranes were incubated with respective HRP-conjugated (CST) or fluorescently labelled (Biorad) secondary antibodies for 1 h, washed again 3x10 min in TBS-T and developed on a ChemiDoc gel imaging system (Biorad) using the respective channels. HRP-conjugated blots were incubated with Immobilon Western Chemiluminescent HRP Substrate (Millipore).

Immunoprecipitation

Cell lysates were adjusted to 1 μg/μl in lysis buffer. Either GFP-trap beads (ChromoTek) or Anti-FLAG-M2-Affinity agarose resin (SigmaAldrich) was equilibrated with lysis buffer. 300-500 μg of total protein was added to 10-15 μl of beads (50% slurry) and incubated for an hour at 4°C under agitation. Centrifugation steps at 200xg were done at 4°C for 2 minutes. Supernatant (flowthrough) was separated from beads, and beads were washed 3-5 times in lysis buffer. Proteins were eluted in lysis buffer containing Laemmli SDS sample buffer by boiling at 95°C for 5 minutes.

Antibodies

Antibodies were purchased from Thermo Fisher (Alpha tubulin, MA1-80189; rat-HRP, 31470; B-RAF, 702187), Abcam (panRAS, ab206969; HIF1α, ab1), Sigma (K-RAS4B, WH0003845M1; Flag-HRP, A8592-.2MG; GFP, 11814460001), CST (GAPDH, 2118S; ERK1/2, 9102S; phospho ERK1/2 (T202/Y204), 9106S; MEK1/2, 4694S; phospho MEK1/2 (S221), 2338S; AKT, 9272S ; phosphor AKT (S473), 12694S; phospho EGF receptor (Y1068), 3777; c-myc, 5605; rabbit-HRP, 7074S; mouse-HRP, 7076S), SantaCruz (EGF receptor, sc-03-G) and Bio-Rad (rabbit starbright 700, 12004161). Primary antibodies were generally used in 1:1,000 dilutions in 5% milk TBS-T, apart from RAS (1:500), and GAPDH & alpha-tubulin (1:5,000). Secondary antibodies were used in a 1:5,000 dilution in 5% milk TBS-T. Other primary antibodies recognizing different RAS species were obtained from Proteintech (N-RAS, 10724-1-AP; H-RAS, 18295-1-AP; K-RAS2B, 16155-1-AP; K-RAS2A, 16156-1-AP) and Invitrogen (H-RAS, PA5-22392; K-RAS, 415700). Antibodies for immunofluorescence were purchased from MBL/Caltag Medsystems (GFP, 598), Abcam (ATPB, ab14730), BD Biosciences (P120 Catenin, 610133), Sigma (Flag-M2, F1804) and Thermo Fisher (AlexaFluor488 [donkey anti-rabbit], A21206; AlexaFluor594 [goat anti-mouse], A11005).

Immunofluorescence

Cells were seeded in a 12-well dish onto cover slips and grown over night. The next day, cells were washed twice in PBS and fixed for 10 minutes in 4% formaldehyde/PBS (Sigma). Coverslips were washed in DMEM (Gibco) containing 10 mM HEPES followed by a 10 min incubation. Coverslips were washed in PBS and permeabilised for 3 min in either 0.2% NP-40/PBS or 0.2% Triton X-100/PBS. Coverslips were washed twice in PBS and blocked for 15 min in 3% BSA (Sigma) in PBS. Primary antibody incubation was done for 1-2 h at room temperature at appropriate antibody dilutions in blocking solution. Residual antibody was washed away in 0.2% Tween/PBS (3x10 min). Secondary antibody incubation was done for 30 min at 1:300 antibody dilution in the dark. The same wash steps were repeated, but the first wash contained DAPI (0.5–1 μg in 10 ml, SigmaAldrich). Finally, coverslips were dipped in water, air dried and mounted on slides with Vectashield (Vector Laboratories). Fluorescence signals were analysed on a Deltavision Widefield microscope (GE). Images were deconvolved using the default settings of softWoRx Imaging software and further analysed using OMERO (Allan et al., 2012).

Cell Proliferation Assays

After trypsinization, live cell numbers were determined in a Neubauer haemocytometer in the presence of trypan blue. Cell numbers were adjusted to 5000 cells per ml in the respective growth medium. 5000 cells were added per well of a 12-well dish, and each line was grown in triplicates. After 7 days, relative cell numbers were determined by crystal violet staining. In short, cells were washed in PBS, fixed for 5 min in fixing buffer (10% methanol, 10% acetic acid), washed in PBS again and incubated for 30-60 min in crystal violet solution (0.5% crystal violet in 20% methanol). Plates were dipped in tap water to remove stain and air dried overnight. Plates were scanned on a Licor Odyssey using the 700 nm channel. Subsequently, 1 ml methanol was added to each well and plates were incubated shaking for 30 min. Depending on the colour of 1 set of cells, 100-200 μl of supernatant was loaded in triplicate on a 96-well plate and absorbance at 570 nm was measured in an Epoch microplate spectrophotometer (BioTek). Values were normalized to the untreated sample and a one-way ANOVA analysis with Dunnett's multiple comparisons test was done.

Flow Cytometric Analysis

Cells were trypsinized, washed and resuspended in PBS containing 1% FBS. Cells were then analysed on a FACS Canto II flow cytometer. Cells were analysed with the following gating strategy: (i) cells: in a plot of FSC-A vs. SSC-A, a gate was drawn surrounding the major population of cells, removing debris and dead cells. (ii) single cells: in a plot of FSC-A vs. FSC-W, a gate was drawn around an area corresponding to single cells. (iii) in the 'single cells' population on a GFP-A vs. PE-A plot a gate was drawn around GFP-positive cells in A549^{GFPKRAS} sample, using WT A549 cells as a negative control. Gates (i) and (ii) were adjusted to the individual cell lines. Gate (iii) was kept unchanged within an experiment.

Sample preparation for Tandem Mass Tag (TMT) Labelling

Transduced and selected A549 cells were processed for TMT labelling as described previously (Tovell et al., 2019). In short, samples were lysed in 8 M Urea and 50 mM Ammonium bicarbonate containing buffer, cleared after benzonase treatment, reduced with 5 mM DTT at 45°C for 30 min, alkylated with 10 mM iodoacetamide at room temperature in the dark for 20 min), quenched by addition of 5 mM DTT, digested with Lys-C (1:200 (w/w), Lys-C/protein) for 4 h at 30°C, diluted with 50 mM Ammonium bicarbonate to 1.5 M final

Urea concentration, followed by trypsin digestion (1:50 (w/w), trypsin/protein) at room temperature overnight. 1% TFA was added to stop the digestion. The acidified digest samples were desalted on 200 mg Sep-PAK tC18 cartridges, and the eluents were dried by using speed vacuum centrifugation (Thermo). Tandem Mass Tag labelling was performed according to the manufacturer's protocol using the TMT Labelling Kit (Thermo, 90110). The complete labelled samples were then mixed and fractionated with high pH reverse phase C18 chromatography using an Ultimate 3000 high-pressure liquid chromatography system (Dionex) at a flow rate of 569 μl/min using two buffers: A (10 mM ammonium formate, pH 10) and B (80% ACN, 10 mM ammonium formate, pH 10). Briefly, the desalted TMT labelled peptides were resuspended in 200 μL of buffer A (10 mM ammonium formate, pH10) and fractionated on a C18 reverse phase column (4.6 × 250 mm, 3.5 μm, Waters) with a gradient as follows: 3% B to 12.5 % B in 10 min, 12.5% to 40% buffer B in 45 min, 40% B to 60% B in 25 min, 60% B to 80% B in 10 min, 80% B to 100% B in 2.5 min, 100% for 5 min, ramping to 3% B in 2.5 min and then 3% for 10 min. A total of 90 fractions were collected and then concatenated into 30 fractions, which were further desalted over C18 StageTips and speed vacuum dried prior to LC-MS/MS analysis.

LC-MS/MS Mass Spectrometry

LC-MS/MS analysis was done as described previously (Tovell et al., 2019), with a Thermo Dionex Ultimate 3000RSLC Nano liquid chromatography instrument. Peptides were quantitated by Nanodrop and the sample was dissolved in 0.1% formic acid. 1 μg of each fraction was loaded on C18 trap column with 3% ACN/0.1% TFA at 5 ul/min flow rate. Peptides were separated over an EASY-Spray column (C18, 2μm, 75μm x 50cm) with an integrated nano electrospray emitter (flow rate 300nl/min). Peptide separation was done over 180 min with a segmented gradient: the first 10 fractions starting from 5%∼30% buffer B in 125 min (Note: the middle 10 fractions starting from 7% and the last 10 fractions starting from 10%), 30%∼45% buffer B in 30 min, 45%∼95% buffer B for 5 min, followed by a 5 min 95% B. Eluted peptides were analysed on an Orbitrap Fusion Lumos (Thermo Fisher Scientific, San Jose, CA) mass spectrometer. Spray voltage was set to 2 kV, RF lens level was set at 30%, and ion transfer tube temperature was set to 275 °C. The Orbitrap Fusion Lumos was operated in positive ion data-dependent mode with synchronous precursor selection (SPS)-MS3 analysis for reporter ion quantitation. The mass spectrometer was operated in data-dependent Top speed mode with 3 seconds per cycle. The full scan was performed in the range of 350–1500 m/z at nominal resolution of 120 000 at 200 m/z and AGC set to $4x10^5$ with maximal injection time 50 ms, followed by selection of the most intense ions above an intensity threshold of 5000 for collision-induced dissociation (CID)-MS2 fragmentation in the linear ion trap with 35% normalized collision energy. The isolation width was set to 0.7 m/z with no offset. Dynamic 6 exclusion was set to 60 seconds. Monoisotopic precursor selection was set to peptide, maximum injection time was set to 50 msec. Charge states between 2 to 7 were included for MS2 fragmentation. The top 5 fragment ions from each MS2 scan was notched out for MS3. The MS3 scan were performed with an isolation width of 2 m/z in the quadrupole, normalised HCD collision energy of 65% and analysis of fragment ions in the orbitrap using 50 000 resolving power with auto normal range scan from m/z 100 to 500 and AGC target of $5x10^4$. The maximal injection time for MS3 scan was set to 86 ms.

LC-MS/MS Data Analysis

LC-MS/MS data analysis was done as described previously (Tovell et al., 2019). All acquired LC-MS data were analysed using Proteome Discoverer software v.2.2 (Thermo Fisher Scientific) with Mascot search engine. Maximum missed cleavages for trypsin digestion was set to 2. Precursor mass tolerance was set to 20 ppm. Fragment ion tolerance was set to 0.6 Da. Carbamidomethylation on cysteine (+57.021 Da) and TMT-10plex tags on N termini as well as lysine (+229.163 Da) were set as static modifications. Variable modifications were set as oxidation on methionine (+15.995 Da). Data were searched against a complete UniProt Human Proteome (Reviewed 20,143 entry downloaded at Nov 2018). Peptide spectral match (PSM) error rates with a 1% FDR were determined by target-decoy strategy coupled to Percolator modelling of true and false matches. Both unique and razor peptides were used for quantitation. Reporter ion abundances were corrected for isotopic impurities based on the manufacturer's data sheets. Reporter ions were quantified from MS3 scans using an integration tolerance of 20 ppm with the most confident centroid setting. Signal-to-noise (S/N) values were used to represent the reporter ion abundance with a co-isolation threshold of 50% and an average reporter S/N threshold of 10 and above required for quantitation from each MS3 spectra to be used. The S/N value of each reporter ions from each PSM were used to represent the abundance of the localised phosphorylation sites. The precursor spectra with higher than 25% co-isolation were further manually checked. The total peptide amount was used for the normalisation. Protein ratios were calculated from medians of summed sample abundances of replicate groups. Standard deviations were calculated from three biological replicate values. The standard deviation of three biological replicates lower than 25% were used for further analyses. To determine the significant differences between different treatments, ANOVA model was used for statistical significance analysis.

QUANTIFICATION AND STATISTICAL ANALYSIS

Statistical analysis was done using GraphPad Prism. For comparison of two groups, unpaired, two-tailed t-tests were performed. For comparison of more than two groups an Ordinary ANOVA with post-hoc Dunnett's multiple comparisons test was performed. A p-value < 0.05 was considered statistically significant. Sample sizes are indicated in the respective figure legend.

Cell Chemical Biology, Volume *27*

Supplemental Information

Targeting Endogenous K-RAS for Degradation through the Affinity-Directed Protein Missile System

Sascha Röth, Thomas J. Macartney, Agnieszka Konopacka, Kwok-Ho Chan, Houjiang Zhou, Markus A. Queisser, and Gopal P. Sapkota

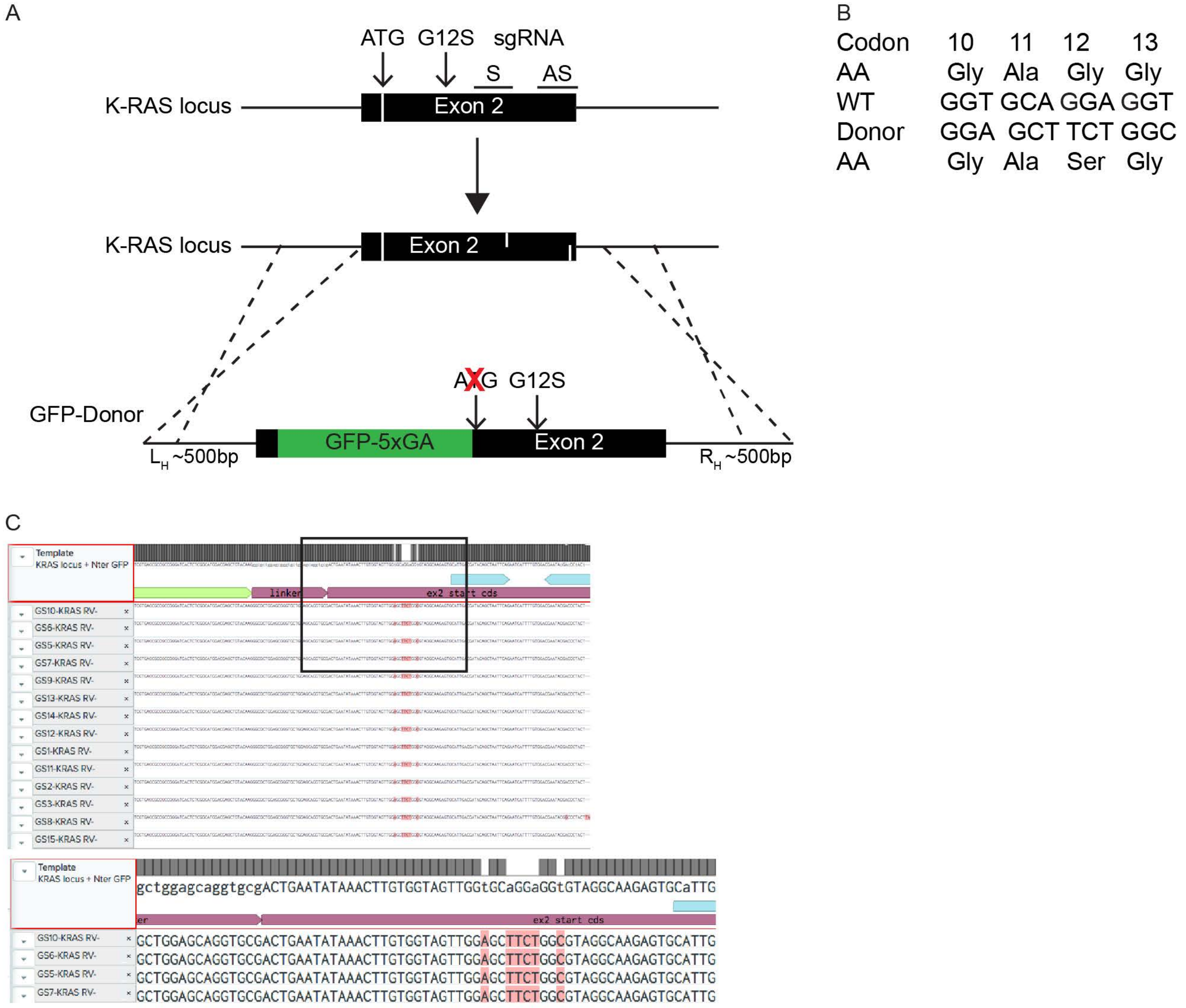

Figure S1 - Characterization of A549^{GFPKRAS} - Related to Figure 1

(A) Schematic representation of the CRISPR/Cas9 strategy used for A549 cells. Two plasmids encoding sgRNA sequences targeting the K-RAS locus on exon 2 were co-expressed with Cas9-D10A, to create two nicks in K-RAS complementary strands for a double stranded break. A donor plasmid consisting of GFP cDNA sequence without the stop codon followed by a GAGAGAGAGA linker flanked by Left and Right homology arms (L_H and R_H, respectively) was designed and co-transfected to allow homologous recombination for insertion of the GFP-5xGA tag onto the native K-RAS locus at the start codon. Consequently, the start codon of K-RAS was eliminated. (B) The indicated silent mutations on sgRNA target codons (10-13) were introduced in the donor sequence to block subsequent dsDNA breaks following integration of the donor on K-RAS locus. (C) A screenshot of DNA sequence analysis from Benchling of the resulting GFP-positive clone. Top: 14 DNA sequence files were aligned against the predicted WT RAS gene locus sequence with the GFP-fusion (indicated in light green) and the 5xGA linker. The box indicates area of the image that is magnified below. The magnified area shows DNA sequence alignment of the A549^{GFPKRAS} cell line at the site of the G12S mutation, which also shows silent mutations. The Ab1 files containing the DNA sequence chromatograms are deposited online.

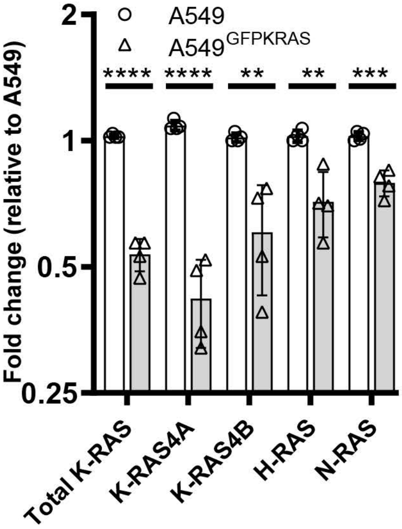

Figure S2 - Analysis of RAS transcript levels in A549 WT and A549^{GFPKRAS} - Related to Figure 1

mRNA was extracted from indicated cell lines and cDNA was synthesised. Fold changes in RAS expression were calculated after qRT-PCR with specific primers between A549^{GFPKRAS} (grey bars) and A549 WT (white bars). Error bars (SD) are shown for n=4. Statistical significance was calculated with an unpaired, parametric, two-tailed t-test. $p<0.01$ = **; $p<0.001$ = ***; $p<0.0001$ = ****.

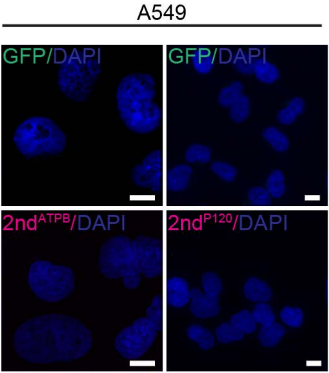

Figure S3 - Negative controls for Figure 1D

Experimental procedure is described in Figure 1D. An additional slide of A549 WT cells (GFP negative) was treated with the secondary antibody used for ATPB or P120. Exposure times are set to be the same as for the positive sample slides in Figure 1D. Scalebar = 10μm

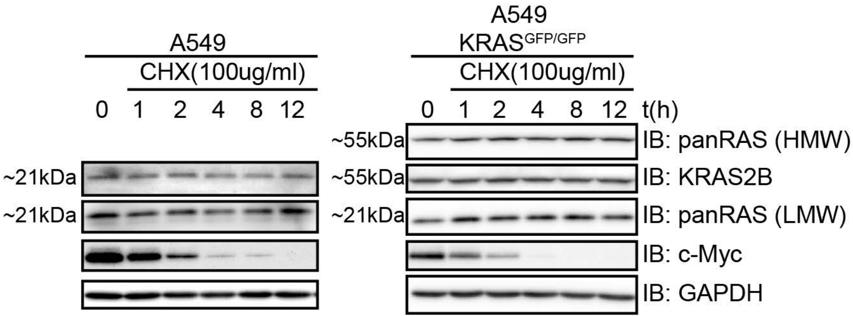

Figure S4 - Representative blots of quantification in Fig. 1E.

Samples were treated as described in Fig. 1E, run on an SDS-PAGE and blotted onto PVDF membrane. The indicated antibodies were used for detection of protein levels. Shown are representative blots for the quantification in Fig. 1E.

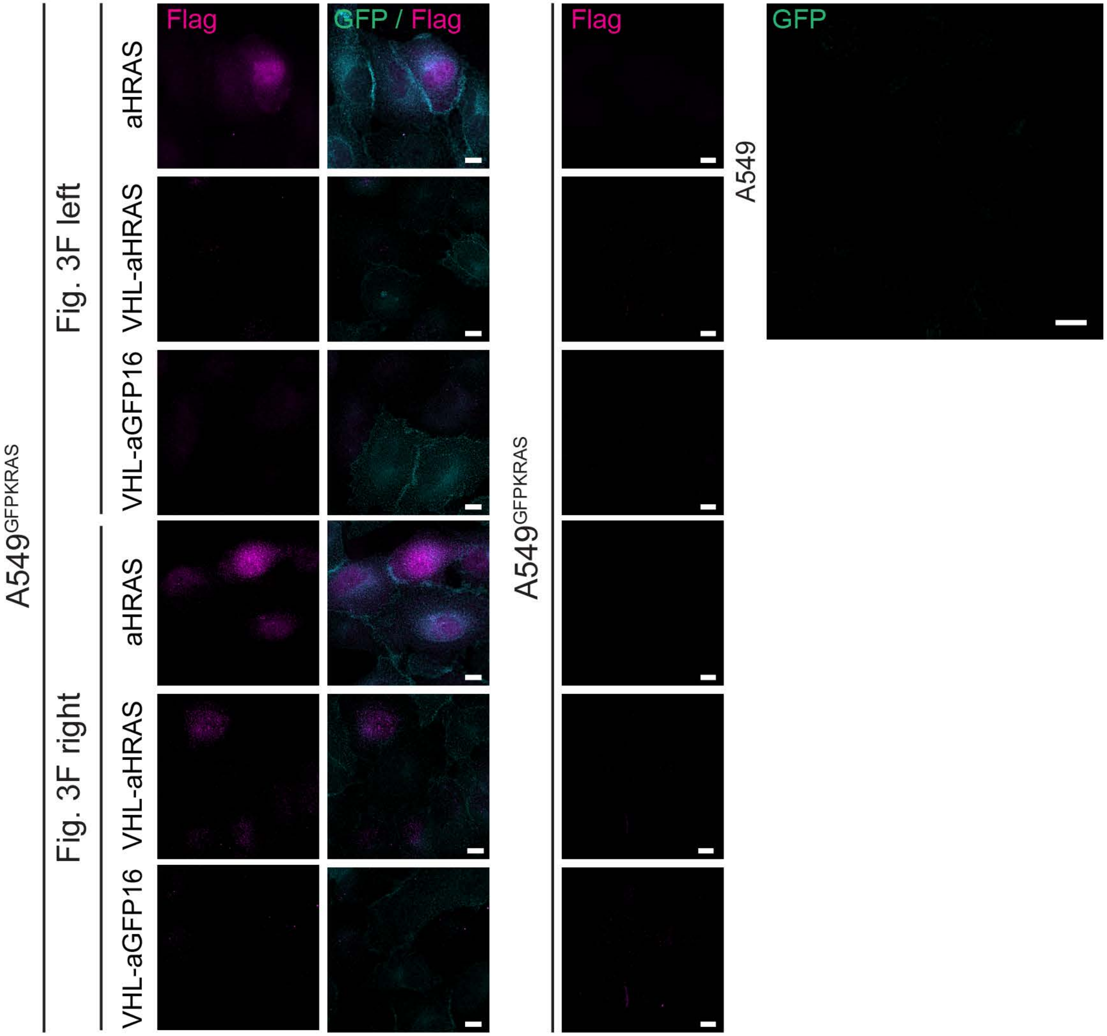

Figure S5 - FLAG signal and controls for Figure 3F.

Slides of Fig. 3F (A549^{GFPKRAS} with or without indicated transductions) were additionally stained with anti-FLAG antibody and secondary stain for detection in the 594 channel (Flag) (2 leftmost columns). The top 3 rows represent the left column of Fig. 3F, the bottom 3 rows the right column of Fig. 3F. Appropriate negative control (A549^{GFPKRAS} cells without a FLAG construct) were stained with the same antibody combination and exposure time (Column 3). Brightness and contrast for individual positive stains were background adjusted for these samples (note that the same negative sample is used for more than one picture). Additionally A549 WT cells were stained with the GFP antibody to act as negative control for GFP signal in main Figure 3F (panel on the right). Scalebar = 10µm.

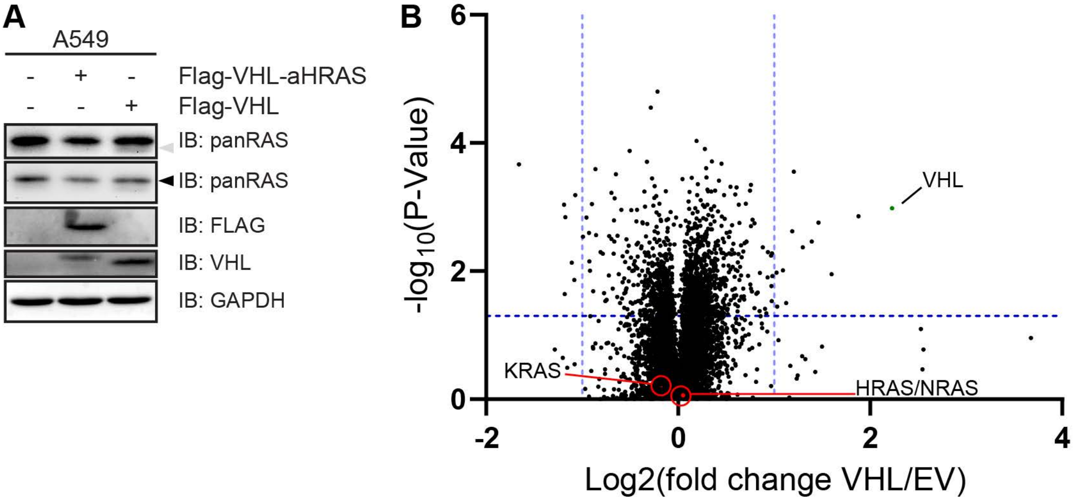

Figure S6 - Proteomic analysis of AdPROM - Related to Figure 4D

(A) Pooled lysates of triplicate A549 cells transduced with and selected for indicated plasmids were subjected to SDS-PAGE and Western blot analysis with indicated antibodies, used for TMT labelling and total proteome analysis. Two images are shown for panRAS detection to highlight K-RAS (black arrowhead) or H-/N/RAS (grey arrowhead) signals. (B) Volcano plot on proteins significantly down- or up-regulated by transduction of A549 cells with Flag-VHL compared to pBabeD empty vector. Horizontal line shows significance level of p=0.05. Vertical lines show 2 fold change. Positions of KRAS, HRAS, NRAS and VHL are indicated.
